# Randomised controlled trial of the short-term effects of osmotic-release oral system methylphenidate on symptoms and behavioural outcomes in young male prisoners with attention deficit hyperactivity disorder: CIAO-II study

**DOI:** 10.1192/bjp.2022.77

**Published:** 2023-01

**Authors:** Philip J. Asherson, Lena Johansson, Rachel Holland, Megan Bedding, Andrew Forrester, Laura Giannulli, Ylva Ginsberg, Sheila Howitt, Imogen Kretzschmar, Stephen M. Lawrie, Craig Marsh, Caroline Kelly, Megan Mansfield, Clare McCafferty, Khuram Khan, Ulrich Müller-Sedgwick, John Strang, Grace Williamson, Lauren Wilson, Susan Young, Sabine Landau, Lindsay D. G. Thomson

**Affiliations:** Social Genetic and Developmental Psychiatry, Institute of Psychiatry Psychology and Neuroscience, King's College London, UK; Department of Biostatistics and Health Informatics, Institute of Psychiatry Psychology and Neuroscience, King's College London, UK; Division of Psychological Medicine and Clinical Neurosciences, Cardiff University, UK; and Department of Forensic and Neurodevelopmental Science, Institute of Psychiatry Psychology and Neuroscience, King's College London, UK; Division of Psychiatry, The University of Edinburgh, UK; Department of Clinical Neuroscience, Karolinska Institute, Sweden; Division of Psychiatry, The University of Edinburgh, UK; and Forensic Psychiatry, NHS State Hospitals Board for Scotland, UK; Forensic Psychiatry, NHS Forth Valley Health Board, UK; Forensic Nursing, NHS State Hospitals Board for Scotland, UK; Forensic Psychiatry, NHS State Hospitals Board for Scotland, UK; Adult ADHD Service, Barnet, Enfield and Haringey Mental Health NHS Trust, UK; Department of Addictions, Institute of Psychiatry Psychology and Neuroscience, King's College London, UK; Psychology Services, Psychology Services Limited, UK; Forensic Psychiatry, NHS State Hospitals Board for Scotland, UK; and Division of Psychiatry, The University of Edinburgh, UK

**Keywords:** Prisoners, methylphenidate, randomised controlled trial, attention-deficit hyperactivity disorders, forensic mental health services

## Abstract

**Background:**

Research has shown that 20–30% of prisoners meet the diagnostic criteria for attention-deficit hyperactivity disorder (ADHD). Methylphenidate reduces ADHD symptoms, but effects in prisoners are uncertain because of comorbid mental health and substance use disorders.

**Aims:**

To estimate the efficacy of an osmotic-release oral system methylphenidate (OROS-methylphenidate) in reducing ADHD symptoms in young adult prisoners with ADHD.

**Method:**

We conducted an 8-week parallel-arm, double-blind, randomised placebo-controlled trial of OROS-methylphenidate versus placebo in male prisoners (aged 16–25 years) meeting the DSM-5 criteria for ADHD. Primary outcome was ADHD symptoms at 8 weeks, using the investigator-rated Connors Adult ADHD Rating Scale (CAARS-O). Thirteen secondary outcomes were measured, including emotional dysregulation, mind wandering, violent attitudes, mental health symptoms, and prison officer and educational staff ratings of behaviour and aggression.

**Results:**

In the OROS-methylphenidate arm, mean CAARS-O score at 8 weeks was estimated to be reduced by 0.57 points relative to the placebo arm (95% CI −2.41 to 3.56), and non-significant. The responder rate, defined as a 20% reduction in CAARS-O score, was 48.3% for the OROS-methylphenidate arm and 47.9% for the placebo arm. No statistically significant trial arm differences were detected for any of the secondary outcomes. Mean final titrated dose was 53.8 mg in the OROS-methylphenidate arm.

**Conclusions:**

ADHD symptoms did not respond to OROS-methylphenidate in young adult prisoners. The findings do not support routine treatment with OROS-methylphenidate in this population. Further research is needed to evaluate effects of higher average dosing and adherence to treatment, multi-modal treatments and preventative interventions in the community.

Attention-deficit hyperactivity disorder (ADHD) is a childhood-onset neurodevelopmental disorder that frequently persists into adulthood. ADHD is defined by a persistent pattern of inattention and/or hyperactivity–impulsivity that interferes with or reduces the quality of functioning in daily life.^1^ Associated problems include educational and occupational failure, transport accidents and the development of antisocial behaviour and criminality.^2^ A further source of impairment is coexisting mental health disorders, such as anxiety, depression, personality disorder and substance misuse.^3^ Adults with ADHD often struggle with mental health symptoms such as a mental and physical restlessness, emotional dysregulation and low self-esteem,^2^ and are more likely to have co-occurring neurological conditions, such as dyslexia and dyspraxia.^4^

Prevalence of ADHD among prisoners is estimated to be around 20–30%, regardless of age and gender,^5^ compared with a population prevalence of 5–7% in children^6^ and 2–3% in adults.^3^ Recommended first-line medications are stimulants, including methylphenidate and lisdexamphetamine, which reduce the core symptoms of inattention and hyperactivity–impulsivity,^7^ as well as emotional dysregulation,^8^ with improvements in other areas of mental health and daily life.^9^ Psychosocial interventions reduce coexisting problems, including poor social skills and conduct problems.^10^

Currently, there is limited information from randomised controlled trials (RCTs) on the efficacy of stimulants for ADHD in prisoners or populations with high levels of comorbid mental health and drug use disorders.^11–13^ The current situation is one of uncertainty among clinicians around the validity of the diagnosis and value of stimulants in prisoners meeting the criteria for ADHD. Significant levels of inattentive, overactive or impulsive behaviour in young male adult prisoners might be better explained by complex post-traumatic stress disorder, personality disorder, drug and alcohol use, other neurodevelopmental disorders or traumatic brain injuries. Stimulant medications may also worsen pre-existing mental health conditions or have reduced efficacy in the prison population because of comorbid mental health or drug use disorders. Treatment trials of methylphenidate in ADHD with co-occurring drug misuse find generally small or non-significant effects on reducing ADHD symptoms.^14^

This study followed a single-arm, open-label study, investigating change in outcomes after treatment with osmotic-release oral system methylphenidate (OROS-methylphenidate) in 121 young male offenders aged 18–25 years.^15^ A large pre–post reduction was observed for investigator-rated ADHD symptoms, with a standardised mean difference (SMD) of >2, reflecting within-patient change over time, with no comparator group. A large effect (SMD = 2.1) was also reported from a small (*n* = 30) RCT of Swedish prisoners, using the same outcome measure.^16^ Large-scale pharmaco-epidemiological studies suggest that treatment of ADHD with methylphenidate reduces criminal convictions,^17^ and violent behaviour on release from prison.^18^ These studies have greater ecological validity, but cannot distinguish pharmacological from non-pharmacological effects. Thus, the existing literature suggests moderate-to-large effects of methylphenidate on reducing ADHD symptoms in offender populations.

The primary objective was to investigate the efficacy of OROS-methylphenidate (over-encapsulated prolonged-release methylphenidate hydrochloride capsules) in reducing ADHD symptoms in young male prisoners, aged 16–25 years, who meet the DSM-5 diagnostic criteria for ADHD. Secondary objectives were the assessment of effects on a wider range of mental health and behavioural outcomes. We further investigated, as putative moderators, a history of childhood trauma, symptoms of borderline personality disorder, and reactive and proactive aggression scores, and whether improvements in secondary behavioural outcomes (antisocial behaviour and educational engagement) could be explained by improvements in ADHD symptoms or emotional dysregulation.

## Method

### Study design

We conducted an 8-week, parallel-arm, randomised placebo-controlled trial of OROS-methylphenidate compared with placebo. Participants were recruited from HM Prison Isis Young Offender Institution (YOI: Oxleas NHS Foundation Trust, London) and HM Prison Polmont YOI (NHS Forth Valley, Scotland). The authors assert that all procedures contributing to this work comply with the ethical standards of the relevant national and institutional committees on human experimentation and with the Helsinki Declaration of 1975, as revised in 2008. All procedures involving human participants were approved by the East of England, Essex, Research Ethics Committee (reference 16/EE/0117). The trial was registered with the European Union Drug Regulating Authorities Clinical Trials Database (EudraCT; number 2015-004271-78) and the ISRCTN Registry (number ISRCTN16827947). The database was locked on 27 August 2019. The protocol is previously published.^19^

### Participants

Informed written consent was obtained for screening with the Barkley Adult ADHD Scale (BAARS) and the Diagnostic Interview for Adult ADHD (DIVA 2.0). A psychiatrist trained in ADHD confirmed the research diagnosis and obtained written consent for the trial.

Inclusion criteria were meeting the DSM-5 ADHD criteria, male gender, aged 16–25 years at the time of consent for screening, and fluency in English. Exclusion criteria were lacking capacity to give informed consent; IQ of <60; serious risk of violence to the researcher; current major depression, psychosis or mania/hypomania; history of bipolar disorder or schizophrenia; medical contraindication to the use of stimulants; drug-seeking behaviour or craving; and currently prescribed ADHD medication.

### Randomisation and masking

Participants were randomly assigned to either OROS-methylphenidate or placebo, using an online system provided by the King's Clinical Trials Unit. Randomisation was at a 1:1 ratio, stratified by prison, using randomly varying block sizes. The randomisation system allocated a study medication kit with a unique pack number. Trial medication was 18 mg over-encapsulated prolonged-release methylphenidate hydrochloride or placebo capsules. All members of the clinical and research teams were blinded to trial arm status. The statistical team were blinded to trial arm status until all planned analyses had been completed. The primary Connors Adult ADHD Rating Scale (CAARS-O) outcome measure was completed by a trained research investigator at the end of weeks 5 and 8, who was not involved in dose titration.

### Procedures

Medication was started within 7 days, but usually within 2–3 days, of randomisation. Medication was given to participants daily and intake was observed. Treatment with OROS-methylphenidate or placebo started at one capsule per day for 1 week. The number of capsules was then increased following weekly assessments with a trial psychiatrist at the end of weeks 2, 3 and 4, to a maximum dose of four capsules per day. Titration upward was continued unless all 18 ADHD symptoms were scored as negligible (0 or 1 on the CAARS-O scale), there were unacceptable adverse effects or participants objected to an increase. The dose at the end of 5 weeks was maintained for the final 3 weeks of the trial.

Research assessments were completed before randomisation and at the end of weeks 1, 2, 3, 4, 5 and 8. Assessments at the end of weeks 1, 2, 3 and 4 were conducted by a trial psychiatrist titrating the dose of the trial medication, and consisted of administering the CAARS-O and Adverse Events Scale (AES), and measuring heart rate and blood pressure. Assessments at the end of weeks 5 and 8 were conducted by a researcher not involved in the titration process, to reduce potential bias from psychiatrists engaged in the titration process, apart from the Clinical Global Impressions Scale (CGI). Participants were informed that participation in the trial would not influence their status or length of the prison sentence. Withdrawal from the study was defined as withdrawal from taking the trial medication and from providing further follow-up assessment data.

### Outcomes

Outcomes measures are listed in Table 1. The primary end-point was the level of ADHD symptoms, measured by the investigator-rated CAARS-O at 8 weeks after treatment initiation. Thirteen secondary outcomes at week 8 included measures of emotional dysregulation (Wender–Rheimherr Adult Attention Deficit Disorder Scale (WRAADDS) and Affective Reactivity Index Self-Report (ARI-S)), common psychopathology (Brief Symptom Inventory (BSI) and Clinical Outcomes in Routine Evaluation – Outcome Measure (CORE-OM)), mind wandering (Mind Excessively Wandering Scale (MEWS)), attitudes toward violence (Maudsley Violence Questionnaire (MVQ)), global impression of therapeutic effect (CGI therapeutic effect), behavioural reports from prison and educational staff (Modified Overt Aggression Scale prison officer rated (MOAS-P) and education staff rated (MOAS-E); and Behaviour Report Card prison officer rated (BRC-P) and education staff rated (BRC-E)) and the number of critical incidents and education sessions attended reported in prison records. In addition, blood pressure, heart rate and common adverse events were recorded.

**Table 1:** Schedule of trial measures

Measure	Description	Rater	Screen	Baseline	Week (post-randomisation)					
					1	2	3	4	5	8
Screening measures										
Barkley Adult ADHD Rating Scale	Screening for ADHD symptoms	Self-report	X							
Diagnostic Interview for ADHD in Adults	ADHD assessment	Investigator	X							
Baseline-only measures										
Demographics	Demographic data	Health records		X						
Mini-International Neuropsychiatric Interview (MINI 7.0.1)	Comorbid mental health disorders	Investigator		X						
Weschler Abbreviated Scale of Intelligence	IQ	Investigator		X						
Alcohol Use Disorders Identification Test – Concise	Alcohol use	Self-report		X						
National Institute on Drug Abuse Quick Test	Drug use	Self-report								
Childhood Trauma Questionnaire	Childhood trauma	Self-report		X						
Zanarini Rating Scale for Borderline Personality Disorder	Borderline personality disorder	Self-report		X						
Reactive–Proactive Aggression Questionnaire	Reactive proactive aggression	Self-report		X						
Weiss Conduct Disorder Scale	Conduct disorder	Self-report		X						
Outcome measures										
Connors Adult ADHD Rating Scale for Observers	ADHD symptoms	Investigator		X	X	X	X	X	X	O
Wender–Rheimherr Adult Attention Deficit Disorder Scale	Emotional dysregulation	Investigator		X					X	O
Affective Reactivity Index – Self-Report	Irritability	Self-report		X					X	O
Mind Excessively Wandering Scale	Spontaneous mind wandering	Self-report		X					X	O
Brief Symptom Inventory	General psychopathology	Self-report		X					X	O
MINI checklist	MINI 7.0.1 symptom checklist	Investigator		X						X
Clinical Outcomes in Routine Evaluation – Outcome Measure	Symptoms of psychological distress	Self-report		X						O
Maudsley Violence Questionnaire	Attitudes toward violence	Self-report		X					X	O
Clinical Global Impressions Scale severity score	Clinical Global Impressions severity subscale	Investigator		X					X	O
Clinical Global Impressions Scale therapeutic response score	Clinical Global Impressions therapeutic outcome subscale	Investigator							X	X
Modified Overt Aggression Scale – prison officer rated	Modified Overt Aggression Scale – prison	Prison officer		X						O
Behaviour Report Card – prison officer rated	Behaviour Report Card – prison	Prison officer		X						O
Modified Overt Aggression Scale – education staff rated	Modified Overt Aggression Scale – education	Education staff		X						O
Behaviour Report Card – education staff rated	Behaviour Report Card – education	Education staff		X						O
Critical incidents	Number of behavioural incidents (adjudications) reported	Prison records		X						O
Education sessions	Number scheduled	Prison records Prison records		X						X
	Number attended			X						O
Medication	Dose prescribed	Health records			X	X	X	X	X	X
	Number of capsules taken	Health records			X	X	X	X	X	X
Adverse Events Scale	Adverse Event Scale	Investigator		X	X	X	X	X	X	X
Heart rate and blood pressure	Heart rate/blood pressure	Investigator		X	X	X	X	X	X	X
Height	Height	Investigator		X						
Body mass index	Body mass index	Investigator		X					X	X

O indicates that the measure was a primary or secondary trial outcome measure. ADHD, attention-deficit hyperactivity disorder.

### Statistical analysis

Sample size was estimated with the *t*-test function in G*Power (Windows, version 3) to compare the means of the treatment groups. For 90% power at the 5% significance level, 142 participants are needed to detect a standardised effect of SMD = 0.55. Assuming a standard deviation of 9.1, reported from the open-label pilot study of OROS-methylphenidate in HM Prison Isis YOI,^15^ this translates into a treatment difference of 5.0 points. This is consistent with the results of a recent meta-regression analysis of methylphenidate on ADHD symptoms in adults, which estimated an average SMD of 0.49 (95% CI 0.08–0.64) reflecting a clinically important change.^7^ Assuming a loss to follow-up of 25%, we set the target sample size at 200.

A statistical analysis plan (SAP) was developed and signed before database lock. Analyses followed the intention-to-treat (ITT) principle, including all participants in the groups to which they were randomised.

Withdrawal from treatment was found to predict missing primary outcome data. To accommodate such a missing at random process and avoid bias, we used multiple imputation, which consists of an imputation and an analysis step. For the primary outcome, the analysis model was a regression model that contained CAARS-O score at 8 weeks as the dependent variable, and trial arm and CAARS-O score at baseline and prison site (randomisation stratifier) as explanatory variables. For secondary outcomes, a similar modelling approach was employed based on respective generalised linear analysis models. Binary secondary outcomes such as any aggressive events reported by prison staff were analysed with logistic regression, and count outcomes such as critical incidents were analysed with a negative binomial model. The SAP also contained a set of planned moderator and mediator analyses, and a set of pre-specified sensitivity analyses. Further details of the SAP analyses can be found in Supplementary Appendix 1 available at https://doi.org/10.1192/bjp.2022.77. All analyses were carried out in Stata (Windows, version 15.1).

## Results

**Fig. 1:**
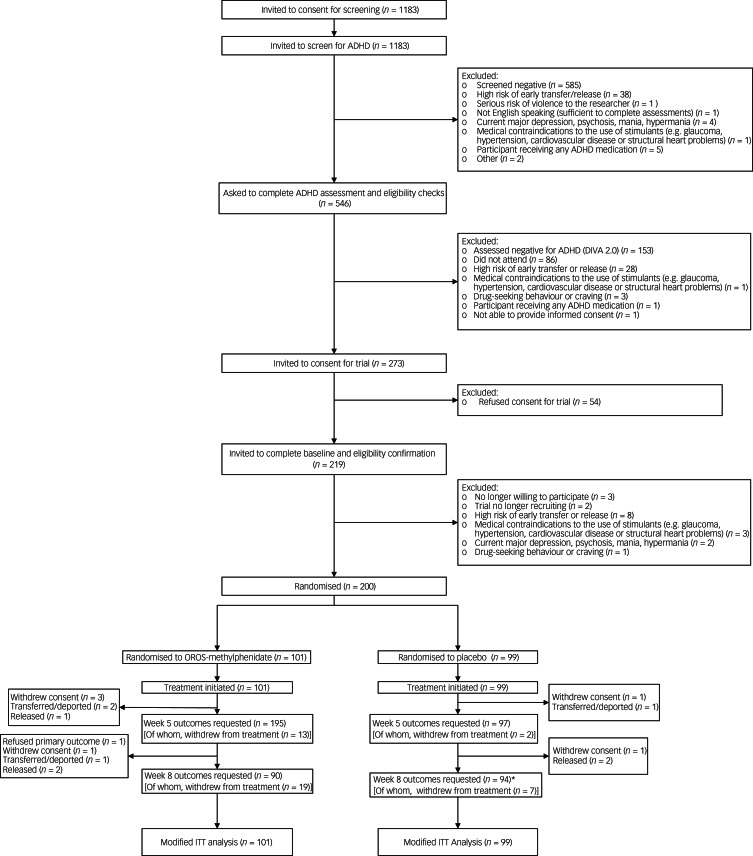
Consolidated Standards of Reporting Trials (CONSORT) trial diagram. *Two participants in the placebo arm were transferred to an accessible prison and the outcomes were collected from those persons. Outcomes were not collected from any other participants labelled as transferred, deported or released. ADHD, attention-deficit hyperactivity disorder; ITT, intention to treat; OROS, osmotic-release oral system.

Figure 1 shows the Consolidated Standards of Reporting Trials (CONSORT) diagram for this trial, including the reasons for exclusion from the trial at each stage.

Participants were recruited from 17 October 2016, when all male prisoners aged 16–25 years at HM Prison Isis YOI and HM Prison Polmont YOI were invited to consent to be screened. The last patient was randomised on 2 April 2019. A total of 1183 prisoners were screened, 432 completed diagnostic assessments with DIVA 2.0, 279 met the DSM-5 diagnostic criteria for ADHD and 219 signed consent for participation in the trial. Of the 200 randomised participants, outcome data at week 8 was obtained for 90 participants prescribed OROS-methylphenidate and 94 taking placebo.

By week 5, participants were titrated to an average of 2.99 and 3.41 daily tablets in the OROS-methylphenidate and placebo arms, respectively. The proportion of prescribed medication taken was 47.2% and 58.4%, respectively (Supplementary Tables 11 and 12). Low adherence to treatment was because of days when participants did not turn up for their medication at the pharmacy, but the entire dose was taken when received.

Baseline demographic and clinical characteristics of the randomised participants are summarised in Tables 2 and 3, and were similar between the two groups.

**Table 2:** Summaries of categorical demographic and baseline-only variables, by trial arm and overall

Item name	Category name	OROS-methylphenidate		Placebo		Overall	
		*N*	*n* (%)	*N*	*n* (%)	*N*	*n* (%)
Site	Isis	101	58 (57.4)	99	57 (57.6)	200	115 (57.5)
	Polmont		43 (42.6)		42 (42.4)		85 (42.5)
Ethnicity	White (White British, White Irish, White other),	101	64 (63.4)	99	61 (61.6)	200	125 (62.5)
	other (Asian, other mixed, other, Black African and White, Black Caribbean and White),		19 (18.8)		10 (10.1)		29 (14.5)
	Black (Black African, Black Caribbean, other Black)		18 (17.8)		28 (28.3)		46 (23.0)
Education	No qualifications	101	42 (41.6)	99	37 (37.4)	200	79 (39.5)
	Any qualifications		59 (58.4)		62 (62.6)		121 (60.5)
Age of leaving school, years	14 or less	101	26 (25.7)	99	25 (25.3)	200	51 (25.5)
	Aged 15		32 (31.7)		22 (22.2)		54 (27.0)
	Over 15		35 (34.7)		43 (43.4)		78 (39.0)
	Unknown		8 (7.9)		9 (9.1)		17 (8.5)
Employed (including in education)	Unemployed	101	67 (66.3)	99	66 (66.7)	200	133 (66.5)
	Employed		34 (33.7)		33 (33.3)		67 (33.5)
Offence category	Serious violence or sexual	101	15 (14.9)	99	14 (14.1)	200	29 (14.5)
	Assault		25 (24.8)		26 (26.3)		51 (25.5)
	Drug-related		27 (26.7)		30 (30.3)		57 (28.5)
	Burglary or theft		27 (26.7)		20 (20.2)		47 (23.5)
	Other, including possession of weapon, driving and wilful fire raising		7 (6.9)		9 (9.1)		16 (8.0)
Previous ADHD treatment	Yes	101	27 (26.7)	99	20 (20.2)	200	47 (23.5)
	No or unknown		74 (73.3)		79 (79.8)		153 (76.5)
Age when ADHD medication last taken, years	≤13	101	9 (8.9)	99	3 (3.0)	200	12 (6.0)
	≥14		18 (17.8)		12 (12.1)		30 (15.0)
	Unknown		74 (73.3)		84 (84.9)		158 (79.0)

Baseline characteristics	*N*	Mean (s.d.)	*N*	Mean (s.d.)	*N*	Mean (s.d.)
Age (range 16–25 years), years	101	20.6 (1.9)	99	20.8 (1.9)	200	20.7 (1.9)
WASI-II	101	89.9 (13.5)	99	88.9 (12.4)	200	89.4 (13.0)
Body mass index	101	23.7 (3.4)	99	23.7 (3.7)	200	23.7 (3.5)
RPAQ – Proactive [range 0–24]	101	6.8 (5.2)	98	7.6 (5.6)	199	7.2 (5.4)
RPAQ – Reactive [range 0–22]	101	14.1 (4.8)	98	14.6 (5.0)	199	14.4 (4.9)
RPAQ total [range 0–46]	101	20.9 (9.2)	98	22.2 (9.7)	199	21.5 (9.4)
CTQ [range 28–140]	101	54.4 (16.9)	99	54.0 (18.1)	200	48.9 (20.7)
ZAN-BPD [range 0–36]	101	6.9 (5.1)	99	6.3 (4.2)	200	6.6 (4.6)
BSI [range 0–212]	101	52.5 (32.5)	99	52.9 (35.9)	200	52.7 (34.2)

Comorbid disorders	*N*	Number with disorder (%)	*N*	Number with disorder (%)	*N*	Number with disorder (%)
Antisocial personality disorder	101	72 (71.3)	99	77 (77.8)	200	149 (74.5)
Mood disorder (major depression, suicidality, mania, hypomania)	101	19 (18.8)	99	19 (19.2)	200	63 (31.5)
Anxiety disorder (panic, agoraphobia, social anxiety, obsessive–compulsive disorder, PTSD)	101	19 (18.8)	99	19 (19.2)	200	38 (19.0)
Potential problematic alcohol use	101	78 (77.2)	99	71 (71.7)	200	149 (74.5)
Illicit drug use	101	99 (98.0)	99	95 (96.0)	200	194 (97.0)

OROS, osmotic-release oral system; ADHD, attention-deficit hyperactivity disorder; WASI-II, Weschler Abbreviated Scale of Intelligence; RPAQ, Reactive–Proactive Aggression Questionnaire; CTQ, Childhood Trauma Questionnaire; ZAN-BPD, Zanarini Rating Scale for Borderline Personality Disorder; BSI, Brief Symptom Inventory; PTSD, post-traumatic stress disorder.

**Table 3:** Summaries of trial outcomes by trial arm and assessment time point

Outcome measure	Time point	OROS-methylphenidate		z		Overall	
		*N*	Mean (s.d.)	*N*	Mean (s.d.)	*N*	Mean (s.d.)
CAARS-O	Baseline	100^a^	36.4 (9.8)	99	37.2 (8.7)	199	36.8 (9.2)
	Week 1	96	32.4 (9.9)	98	31.2 (11.5)	194	31.8 (10.7)
	Week 2	92	28.8 (11.2)	97	29.6 (11.3)	189	29.2 (11.3)
	Week 3	93	28.3 (11.3)	94	30.3 (12.0)	187	29.3 (11.7)
	Week 4	92	26.0 (12.5)	96	29.1 (11.9)	188	27.6 (12.3)
	Week 5	92	27.5 (12.7)	95	28.8 (11.5)	187	28.2 (12.1)
	Week 8	90	28.0 (11.9)	94	29.3 (11.6)	184	28.7 (11.7)
MEWS	Baseline	101	25.7 (6.7)	99	26.8 (6.2)	200	26.3 (6.5)
	Week 5	92	20.5 (9.3)	95	21.4 (9.4)	187	21.0 (9.3)
	Week 8	90	19.8 (10.0)	94	21.9 (9.2)	184	20.9 (9.6)
BSI	Baseline	101	52.5 (32.5)	99	52.9 (35.9)	200	52.7 (34.2)
	Week 5	92	38.4 (28.6)	95	36.3 (25.3)	187	37.4 (26.9)
	Week 8	88	35.0 (25.1)	93	39.0 (34.1)	181	37.1 (30.0)
WRAADDS	Baseline	101	17.5 (5.7)	99	18.1 (5.6)	200	17.8 (5.7)
	Week 5	92	13.6 (5.8)	95	14.3 (6.5)	187	14.0 (6.2)
	Week 8	90	13.4 (6.1)	94	14.5 (7.0)	184	13.9 (6.6)
ARI-S	Baseline	101	9.3 (3.5)	99	9.3 (3.7)	200	9.3 (3.6)
	Week 5	92	8.2 (3.7)	95	7.6 (4.2)	187	7.9 (3.9)
	Week 8	90	8.2 (4.1)	94	8.0 (4.5)	184	8.1 (4.3)
CORE-OM	Baseline	101	43.5 (13.9)	99	44.8 (15.3)	200	44.2 (14.6)
	Week 8	89	38.0 (12.3)	94	39.0 (13.4)	183	38.6 (12.8)
MVQ	Baseline	101	33.2 (9.4)	99	34.6 (9.9)	200	33.9 (9.6)
	Week 5	92	30.8 (11.2)	94	32.4 (10.9)	186	31.6 (11.0)
	Week 8	90	30.6 (12.5)	94	33.1 (11.7)	184	31.9 (12.1)
CGI therapeutic effect	Week 5	84	10.0 (4.1)	94	10.9 (3.0)	178	10.5 (3.6)
	Week 8	86	10.1 (4.2)	93	10.9 (3.4)	179	10.5 (3.8)

Prison officer–reported outcomes		*N*	Minimum/median/maximum	*N*	Minimum/median/maximum	*N*	Minimum/median/maximum
MOAS-P [range 0–40]	Baseline	101	0/0/15	99	0/0/19	200	0/0/19
	Week 8	88	0/0/24	91	0/0/20	179	0/0/24
BRC-P [range 6–30]	Baseline	101	6/8/24	99	6/8/21	200	6/8/24
	Week 8	88	6/9/25	91	6/8/25	179	6/9/25
Number of critical Incidents	Baseline	101	0/0/6	99	0/0/10	200	0/0/10
	Week 8	97	0/0/8	97	0/0/8	194	0/0/8

Education staff–reported outcomes		*N*	Number (%) in education	*N*	Number (%) in education	*N*	Number (%) in education
Number in any kind of education	Baseline	101	94 (93.1)	99	93 (94.0)	200	187 (93.5)
	Week 8		94 (93.1)		97 (98.0)		191 (95.5)

		*N*	Minimum/median/maximum	*N*	Minimum/median/maximum	*N*	Minimum/median/maximum
MOAS-E [range 0–40]	Baseline	38	0/0/12	45	0/0/10	83	0/0/12
	Week 8	31	0/0/1	37	0/0/2	68	0/0/2
BRC-E [range 6–30]	Baseline	31	13/19/41	36	13/17/40	67	13/19/41
	Week 8	28	13/17/29	36	13/18/48	64	13/18/48
Number of education sessions scheduled	Baseline	101	0/21/123	99	0/25/126	200	0/23/126
Number of education sessions attended			0/15/112		0/19/126		0/17/126
Number of education sessions scheduled	Week 8	94	0/34/146	97	1/32/142	191	0/32/146
Number of education sessions attended			0/23/143		0/22/142		0/23/143
		*N*	Mean (s.d.)	*N*	Mean (s.d.)	*N*	Mean (s.d.)
Proportion of education sessions attended	Baseline	101	0.78 (0.31)	99	0.82 (0.26)	200	0.81 (0.29)
	Week 8	94	0.80 (0.28)	97	0.82 (0.26)	191	0.81 (0.27)

OROS, osmotic-release oral system; CAARS-O, Connors Adult ADHD Rating; MEWS, Mind Excessively Wandering Scale; WRAADDS, Wender–Rheimherr Adult Attention Deficit Disorder Scale; ARI-S, Affective Reactivity Index Self-Report; CORE-OM, Clinical Outcomes in Routine Evaluation – Outcome Measure; BSI, Brief Symptom Inventory; MVQ, Maudsley Violence Questionnaire; CGI, Clinical Global Impressions Scale; MOAS-P, Modified Overt Aggression Scale prison officer rated: BRC-P, Behaviour Report Card prison officer rated; MOAS-E, Modified Overt Aggression Scale education staff rated; BRC-E, Behaviour Report Card education staff rated.

a. CAARS-O reported for 100 cases in OROS-methylphenidate arm because there were >20% missing items (two out of nine) in the hyperactivity–impulsivity subscale for one individual.

Symptom severity was similar for the inattentive and hyperactive–impulsive symptoms, with mean baseline scores of 18.2 and 18.6, respectively. Mean age at randomisation was 20.7 years. A total of 62.5% of participants were White and 37.5% were from other ethnic groups. IQ was below the population mean (IQ = 89.4, s.d. = 13.0). A total of 39.5% had no qualifications, with most having left school before the age of 16 years, and 66.5% were unemployed. The majority of participants (76.5%) had not previously received ADHD medication. Most of the sample met the criteria for antisocial personality disorder (74.5%), problem alcohol use (74.5%) and illicit drug use (97%). However, in additional analyses, we found that few met the criteria for high risk of illicit drug use when using the National Institute on Drug Abuse Quick Screen V1.0 criteria (Supplementary Table 15). Common comorbidities included anxiety (19.0%) and mood (31.5%) disorders. Concomitant medications were prescribed at baseline and throughout the trial for 89 participants, of which 48.3% were non-psychotropic medications and 25.8% were antidepressants.

The primary outcome, CAARS-O score at 8 weeks, was used to estimate the efficacy of treating young male prisoners with OROS-methylphenidate on reducing ADHD symptoms. In both trial arms there was a reduction in ADHD symptoms from baseline to the 8-week outcome (Table 3). There was a greater estimated reduction in CAARS-O scores in the OROS-methylphenidate arm of 0.57 (95% CI −2.41 to 3.56) at 8 weeks, compared with the placebo arm. However, this is a small and non-significant difference, with an SMD of 0.06 (Table 4). The trial arm difference is small even at the upper limit of the confidence interval. To investigate the responder rate, we defined a responder as a 20% reduction in the baseline CAARS-O score. The percentage of responders was 48.3% for the OROS-methylphenidate arm and 47.9% for the placebo arm.

**Table 4:** Estimated trial arm differences for the primary and secondary outcomes at week 8

Trial arm differences for continuous outcomes		OROS-methylphenidate versus placebo (positive differences indicate an improvement in the OROS-methylphenidate compared with placebo arm)			
Measure	Description	Estimated difference^a^	95% CI	Test (d.f.), *P*-value	Standardised difference
CAARS-O	ADHD	0.57	−2.41 to 3.56	*t*(183) = 0.38, 0.71	0.06
MEWS	Mind wandering	1.06	−1.39 to 3.52	*t*(182) = 0.85, 0.39	0.16
WRAADDS	Emotion dysregulation	0.83	−0.83 to 2.48	*t*(175) = 0.98, 0.34	0.15
ARI-S	Irritability	−0.31	−1.37 to 0.75	*t*(176) = −0.58, 0.57	−0.09
CORE-OM	Psychological distress	−0.18	−3.46 to 3.10	*t*(175) = −0.11, 0.91	−0.01
BSI	Common psychopathology	2.46	−4.81 to 9.74	*t*(177) = 0.67, 0.51	0.07
MVQ	Attitudes to violence	1.08	−1.16 to 3.33	*t*(179) = 0.95, 0.34	0.11
CGI therapeutic effect	Impression of therapeutic effect	0.65	−0.46, 1.76	*t*(171) = 1.15, 0.25	Not applicable^b^

		OROS-methylphenidate versus placebo (ratios above 1 indicate an improvement under OROS-methylphenidate)			
Odds ratios or IRRs comparing secondary binary and count outcomes between trial arms	Estimated odds ratio [ln(odds ratio)]	95% CI for odds ratio [95% CI for ln(odds ratio)]	Test (*z*)	*P*-value
MOAS-P	Any act of aggression reported in the week before the week 8 time point (yes/no)	0.57 [−0.56]	0.28–1.15 [−1.26 to 0.14]	−1.56	0.12
		Estimated IRR [ln(IRR)]	95% CI for IRR [95% CI ln(IRR)]	Test (*z*)	*P*-value
BRC-P	Behavioural report score for the week before the week 8 time point	0.95 [−0.06]	0.85–1.06 [−0.16 to 0.06]	−0.98	0.33
Critical incidents	Number of critical incidents recorded across the 8 weeks of the trial	0.75 [−0.28]	0.45–1.25 [−0.79 to 0.23]	1.09	0.28
Education sessions scheduled	Number of any type of education session scheduled across the 8 weeks of the trial	0.98 [−0.02]	0.84–1.14 [−0.17 to 0.13]	−0.28	0.78

OROS, osmotic-release oral system; ADHD, attention-deficit hyperactivity disorder; CAARS-O, Connors Adult ADHD Rating; MEWS, Mind Excessively Wandering Scale; WRAADDS, Wender–Rheimherr Adult Attention Deficit Disorder Scale; ARI-S, Affective Reactivity Index Self-Report; CORE-OM, Clinical Outcomes in Routine Evaluation – Outcome Measure; BSI, Brief Symptom Inventory; MVQ, Maudsley Violence Questionnaire; CGI, Clinical Global Impressions Scale; IRR, incidence rate ratio; MOAS-P, Modified Overt Aggression Scale prison officer rated: BRC-P, Behaviour Report Card prison officer rated.

a. All inferences were derived by multiple imputation as described in the Method section. Each model used *k* = 100 imputations.

b. Differences were standardised by dividing by the baseline SD for relevant variables. CGI therapeutic effects was not recorded at baseline.

At 8 weeks, there were small improvements for the OROS-methylphenidate arm in WRAADDS, MEWS, MVQ, BSI and CGI (therapeutic effects) scores, compared with the placebo arm, but small deteriorations were seen in ARI-S and CORE-OM scores. However, the observed changes were all small, with SMDs <0.2 in all cases, and far from statistical significance. Similarly, there were no significant effects for reports of behavioural problems and attendance of educational sessions from the prison records or reports of behaviour from prison officers (Table 4). Education staff reports of behaviour (MOAS-E and BRC-E) were not analysed because of low information content.

Four sensitivity analyses were conducted for the primary outcome (Supplementary Appendix 2, Section 3.4). Regarding adherence to medication, we defined good adherence as taking prescribed trial medication on at least 75% of the days in which medication was prescribed. Only 83 (41.5%) of the 200 participants in the ITT analysis met this criterion: 34 in the OROS-methylphenidate arm and 49 in the placebo arm. Other sensitivity analyses were largely uninformative and showed no differences from primary ITT analyses.

Baseline moderators were investigated, including borderline personality disorder (Zanarini Rating Scale for Borderline Personality Disorder), childhood trauma (Childhood Trauma Questionnaire) and reactive and proactive aggression scores (Reactive–Proactive Aggression Questionnaire ). None of these putative moderators modified the effect of OROS-methylphenidate on the primary outcome (all *P*-values ≥0.1; Supplementary Table 5).

Mediation analyses investigated the effect of CAARS-O hyperactivity subscores, CAARS-O inattention subscores and WRAADDS emotional dysregulation scores, measured at 5 weeks, on prison officer–reported behaviour (BRC-P) and the number of critical incidents reported in prison records at 8 weeks. Mediation effects were close to zero and not statistically significant in all cases, providing no evidence for these mechanisms of action (Supplementary Table 4).

During the trial, one serious adverse event took place, unrelated to the trial mediation, and was categorised as an important medical event. There was a total of 336 different adverse events reported during the trial (184 OROS-methylphenidate arm, 152 placebo arm). These were similar in frequency between the two arms, apart from appetite loss and depressed mood (Supplementary Table 6).

Expected adverse effects were also followed up systematically at each visit, using the medication AES (Supplementary Tables 7–9). The most common adverse effects in the post-randomisation period that were related to the use of OROS-methylphenidate, compared with the use of placebo, were headache (17.8 *v*. 10.1%), dry mouth (19.8 *v*. 10.1%), sweating (19.8 *v*. 8.1%) and appetite reduction (34.7 *v*. 19.2%).

No trial relevant differences were noted for blood pressure and heart rate (Supplementary Table 10).

### Additional *post hoc* analyses

We conducted a series of additional analyses on the primary outcome, proposed after database lock and review of the findings, to explore possible explanations for the trial findings (Supplementary Appendix 3). No potential explanations could be identified for the absence of an effect of OROS-methylphenidate on ADHD symptoms, compared with placebo. This included adherence to medication, final dose, comorbid disorders, self-reported drug use, diagnostic certainty and history of childhood trauma.

## Discussion

The findings from the primary trial analysis, according to our pre-specified SAP, provided no evidence for any post-treatment difference between treatment arms on reducing ADHD symptoms. Sensitivity analyses on the primary outcome found negligible differences, including a per-protocol analysis of a subgroup with high levels of adherence to the trial medication. No baseline modifiers of the primary outcome were identified. Secondary outcomes on associated mental health, behaviour in the prison and educational outcomes also failed to show any statistically significant differences between the study arms, and any differences were negligible.

The lack of any benefit of OROS-methylphenidate compared with placebo was unexpected. Methylphenidate has been used in numerous RCTs of ADHD in children and adults, and consistently shows moderate-to-large effects on reducing ADHD symptoms,^7^ including prior evidence of effects of methylphenidate in offenders with ADHD.^15,16,18,20^

When comparing the baseline to 8-week outcome score differences for the CAARS-O in the open-label pilot trial, using data from HMP Isis YOI only, we found a greater decrease in ADHD symptoms in the pilot study (25.0) compared with the current trial (11.1). Small differences in the ethnic mix and highest educational level achieved are unlikely to explain such a large difference, suggesting that either rater or reporter bias might have been considerable in the uncontrolled open study (Supplementary Tables 16 and 17). In the current trial, around 50% showed an improvement in the OROS-methylphenidate arm, yet there was a comparable improvement in the placebo group, potentially indicating a significant response to placebo. The placebo and nocebo responses in RCTs of ADHD medications have been investigated by systematic review and meta-analysis, and significant and varied placebo effects on ADHD outcomes identified.^21^ It may be that in an environment impoverished of meaningful interactions with others in a caring role, there was an enhanced placebo effect contributing to the outcome of this study.

The promising results from the earlier small RCT in Sweden had several differences with the current trial. Participants were drug-free for 3 months before the trial and had drug testing throughout the trial. They used a fixed dosage, with rapid titration to 72 mg over 1 week, and there was complete adherence throughout the trial. In contrast, the current trial titrated dose more slowly, the final mean dose was 54 mg and by week 8 only 53% of prescribed trial medication was taken. Participants in the Swedish trial had long-term sentences in a high-security prison, and had agreed to be hosted in a specific wing of the prison, with limited contact with other inmates, during the period of the RCT. Consequently, there were younger inmates declining participation, who prioritised other activities.

To address the potential effect of poor adherence, we conducted a *post hoc* subgroup analysis with only those participants who had complied with treatment, defined as taking prescribed trial medication on at least 75% of the days during the 8-week trial. Only 83 out of 200 (41.5%) of the participants met this criterion. Additional *post hoc* per-protocol analyses were conducted, using different definitions for adherence (Supplementary Appendix 3, Section 16) but negligible differences between trial arms were found in all cases.

In further *post hoc* analyses, we investigated the effect of dose by estimating treatment effects for subgroups with a low (one or two capsules) and high (three or four capsules) final dose (Supplementary Appendix 3, Section 12). However, the difference in the primary outcome between trial arms was greater in the low-dose compared with the high-dose subgroup (Supplementary Appendix 3, Section 15). Nevertheless, it remains possible that high levels of illicit drug use might have led to resistance to the usual effects of methylphenidate in this study. For example, a 24-week RCT of OROS-methylphenidate in 54 released prisoners with ADHD and amphetamine dependence, with dose titration to a maximum of 180 mg per day, found significant drug versus placebo differences on ADHD symptoms.^22^ This raises the possibility that higher dosing might have been needed to see clinical benefits in this population.

Another consideration is diagnostic accuracy. To address this, we conducted an additional subgroup analysis in participants reporting high levels of ADHD symptoms in both the inattentive and hyperactive–impulsive symptoms domains during childhood and adulthood (Supplementary Appendix 3, Section 8). Further analyses investigated subgroups without comorbid mental health or drug and alcohol use disorders that might provide alternative explanations for some of the symptoms of ADHD, and subgroups with and without a history of childhood trauma (Supplementary Appendix 3, Sections 10–13). None of these additional subgroup analyses identified significant differences between the two study arms.

### Limitations

We did not conduct drug testing, raising the possibility that unmeasured use of drugs might have affected the measurement of ADHD symptoms, and included participants with a range of comorbid mental health disorders. We considered that drug testing in the protocol would have led to a less representative sample, whereas we wished to evaluate effects in an ecologically valid sample by including all those that met diagnostic criteria for ADHD. We also did not record a history of traumatic brain injury, which has been reported in 40–60% of prisoners^23^ and might lead to an ADHD-like syndrome with a different aetiology and response to methylphenidate.^24^ Some participants received psychological therapies from healthcare staff, potentially reducing trial arm differences. The relationship between participants and research/prison staff might have influenced participants reports of symptoms and behaviours. The Hawthorne effect,^25^ being aware that you are being observed, might have a strong influence on symptom reports in prison settings, where patients may want to please assessors by reporting improvements. However, there was also no trial arm differences for reports of behaviour from education and prison staff.

Diversion of trial medication to other prisoners could play a potential role, but close monitoring did not identify a diversion problem. Furthermore, there was relatively poor adherence to the trial medication compared with more sedative prescription drugs known to be diverted between prisoners within the prisons. Poor adherence could be related in part to the prison regimes. Although the trial medication was supervised, receiving this was dependent on the individual going to receive their medication at the required times.

Imprisonment itself may also have a negative impact on an individual's mental health, affecting the results. A systematic review^26^ of the influence of the prison environment on the mental health of adult prisoners found four main themes: social (isolation, lack of activity and mental stimulation; bullying, violence and exploitation); emotional (family disconnection), organisational (structure, loss of autonomy, respite and access to health services) and physical aspects (overcrowding). However, aspects such as structure, respite and access to health services may be positive.

The low stimulation of the prison environment may also have affected the outcome measures in this study. This trial investigated medication alone and did not include any prosocial competence training, which has been found to be helpful in treating young adults with ADHD.^27^ It is possible that such interventions are an essential component required for a treatment response in this population, and should be considered in future research.

Further studies of ADHD in incarcerated populations should also consider using a final fixed dose to avoid potential biases during the titration process; prison processes required to maximise adherence to medication; drug testing to account for otherwise unmeasured drug effects; applying more stringent diagnostic criteria, such as including only combined type presentations and only those with a clear informant account of ADHD from childhood; exclusion of those with no directly measurable functional deficits, such as educational performance; and inclusion of experimental measure of ADHD-associated deficits, such as increased activity levels and sustained attention deficits.

In conclusion, this trial is robustly neutral and does not support the use of OROS-methylphenidate in the routine treatment of young male adult offenders meeting the diagnostic criteria for ADHD. The findings are of general relevance to other young adult male prison populations because the sample targeted the most frequent type of offenders, including mainly low-to-medium risk offenders with relatively short sentences. To increase generalisability, we applied a screening approach and kept exclusion criteria to a minimum.

Although the findings do not support the use of OROS-methylphenidate in the routine treatment of ADHD in prisoners, they do not exclude a role for healthcare services in managing prisoners with ADHD. There should be a multidisciplinary and multiagency review of people who present with complex and multiple clinical conditions, including ADHD. Although this framework is already meant to exist for those with serious mental illnesses via the Care Programme Approach in England and Wales, a wider framework including health and social care components may be useful for all prisoners who present with one or more conditions. In line with developments in community psychiatry, treatment for ADHD should be integrated within existing prison mental health teams, and considered alongside the treatment of substance misuse and comorbid mental health and neurodevelopmental conditions. Poor response to methylphenidate in this trial might reflect the severity of ADHD and entrenched behaviours, highlighting the need for improved early detection and prevention of at-risk children.

Future studies of prisoners with ADHD should evaluate the effects of higher doses using fixed dose titration. We cannot rule out the potential effect that the prison environment and trial procedures had on reporting of ADHD symptoms, and therefore recommend that future studies should investigate the treatment of ADHD in offenders in community settings. Future studies should also investigate the effects of a more comprehensive multi-modal approach to treatment, including psychosocial and psychological treatments alongside medication.

## Data Availability

All data-sharing requests should be submitted to the corresponding author, P.J.A.,, for consideration. Access to available anonymised data may be granted following review. The data-set that will be shared will be a pseudo-anonymised data-set, and will not include data on date of birth, initials or prison.

## Supplementary material

For supplementary material accompanying this paper visit https://doi.org/10.1192/bjp.2022.77. click here to view supplementary material
