# Supplementary material for: Randomised controlled trial of the short-term effects of osmotic-release oral system methylphenidate on symptoms and behavioural outcomes in young male prisoners with attention deficit hyperactivity disorder: CIAO-II study
Source: Br J Psychiatry. 2023 Jan;222(1):7–17. doi: 10.1192/bjp.2022.77 (PMC7613969; doi:10.1192/bjp.2022.77)
Supplement: Supplementary file 1 [file S0007125022000770sup001.docx]

**SUMMARY OF THE STATISTICAL ANALYSIS REPORT AND SUPPORTING DOCUMENTS**

**A randomised controlled trial of the short-term effects of OROS-methylphenidate on ADHD symptoms and behavioural outcomes in young male prisoners with attention deficit hyperactivity disorder (CIAO-II)**

| **Content** | **Page(s)** |
| --- | --- |
| **Appendix 1: Statistical Analysis Plan (SAP)** | 3 |
| 1. Primary and secondary objectives | 3 |
| Table 1: Primary and secondary outcome measures at week 8 | 5 |
| 1. Statistical methodology | 6 |
| - 1. Approach used to formally compare the primary and secondary outcomes between the active and placebo arms: | 6 |
| - 1. Need for multiple imputation | 7 |
| - 1. Analysis model | 8 |
| - 1. Imputation model | 8 |
| - 1. Sensitivity analyses | 8 |
| - 1. Mechanisms analyses | 9 |
|  |  |
| **Appendix 2: Results from SAP report** | 10 |
| Table 2: Summaries of clinical baseline variables by trial arm and overall | 10 |
| Figure 1: CAARS-O means by trial arms | 14 |
| Table 3: Responders by trial arm | 14 |
| 3.4. Sensitivity analyses | 15 |
| Table 4: Mediation analyses | 16 |
| Table 5: Formal assessment of moderator effects | 17 |
| Table 6: Adverse events by body system code by trial arm | 17 |
| Table 7: Summaries of the Adverse Events Scale by trial arm and overall | 19 |
| Table 8: Adverse Events Scale detail – numbers and percentage with the symptom – baseline | 19 |
| Table 9: Adverse Events Scale detail – numbers and percentage with the symptom – across the trial period | 21 |
| Table 10 Summaries of vital signs by trial arm and overall | 22 |
| Table 11: Summaries of daily tablets prescribed by week, trial arm and overall | 23 |
| Table 12: Summaries of daily tablets taken by week, trial arm and overall | 23 |
| Figure 2: Weekly mean number of tablets taken by trial arm | 24 |
| Table 13: Concomitant medication at baseline | 24 |
| Table 14: Concomitant medications during the trial | 25 |
| Table 15: Illicit drug use prior to incarceration | 25 |
|  |  |
| **Appendix 3: Additional (post-hoc) investigations** | 26 |
| 1. Introduction | 27 |
| 1. Investigating differences between the open pilot study and the randomised controlled trial | 28 |
| Table 16: Comparison of means for CAARS-O for pilot study and the subset of trial patients at HMP YOI ISIS taking OROS-MPH at least once in any consecutive period of 10 days | 29 |
| Table 17: Comparison of demographic and baseline measures for pilot study (CIAO-I) and the current trial (CIAO-II) | 29 |
| 1. Was the study outcome effected by a systematic change to trial procedures? | 30 |
| Table 18: Descriptive summaries across trial arm | 31 |
| 1. Was the study outcome affected by including less severe cases of ADHD in the trial? | 31 |
| Table 19: Subgroup for those with a CAARS-O raw score 35 or above | 31 |
| 1. Was the study outcome affected by poor diagnostic accuracy in this prison population? | 31 |
| Table 20: Subgroup meeting higher threshold diagnostic criteria for ADHD | 32 |
| 1. Could the study outcome be affected by including participants with different levels of emotional dysregulation? | 32 |
| Table 21: WRAADDS baseline severity compared to CAARS-O score | 33 |
| 1. Could the study outcome be affected by including participants with borderline personality disorder? | 33 |
| 1. Could the study outcome be affected including participants with high levels of childhood trauma? | 33 |
| Table 22: CTQ sub-scale | 34 |
| 1. Could the study outcome be affected by including participants with comorbid disorders? | 35 |
| 1. Could the study outcome be affected by including participants with high levels of drug and alcohol? | 36 |
| Table 23: NIDA scores for each drug class | 37 |
| 1. Could the study outcome be affected by including participants with prior experience of stimulant medication leading to biased reporting of the ADHD symptom response to medication? | 38 |
| 1. Could the study outcome be affected by under-dosing of participants? | 38 |
| Table 24: The number of prescribed capsules at the start of week 5 and week 6 for those ongoing in the trial | 39 |
| 1. Could the study outcome be affected by poor adherence to trial medication? | 39 |
| Table 25: Mean daily tablets prescribed by week of those who were continuing treatment | 40 |
| Table 26: Mean daily tablets taken by week and percentage of prescribed dosage taken | 41 |
| Figure 3: Mean adherence with prescribed trial medication | 42 |
| Table 27: Summaries of mean CAARS-O scores at 5 weeks for the subgroup with good compliance in the first 5 weeks of the trial | 43 |
| 1. Could adverse effects be driving adherence to the trial medication? | 43 |
| Table 28: Adherence to medication | 44 |

**Appendix 1: Summary of Statistical Analysis Plan (SAP)**

1. **PRIMARY AND SECONDARY OBJECTIVES**

The overall aim of the trial is to investigate the effects of OROS-MPH in young male prisoners (age 16-25) meeting DSM-5 diagnostic criteria for ADHD. A full list of outcome measures can be found in Table 1. The following study questions will be addressed:

1. What is the efficacy of OROS-MPH in reducing inattention and hyperactivity-impulsivity in young male prisoners meeting diagnostic criteria for DSM-5 ADHD?
2. What is the efficacy of OROS-MPH in reducing secondary outcomes that are key indicators of behavioural and functional impairments used in the management of young prisoners in the UK? These include emotional dysregulation, antisocial behaviour in the prison, violent attitudes (a measure linked to aggression) and reports of behaviour from prison staff.
3. Are improvements in secondary behavioural outcomes mediated by improvements in ADHD symptoms or emotional dysregulation?
   1. **Primary endpoint**

Outcome measures are listed in Table 1.

The primary endpoint is the level of ADHD symptoms measured on the investigator rated Connors Adult ADHD rating scale (CAARS-O) (8) at 8 weeks post treatment initiation to address the question of efficacy of OROS-MPH on ADHD symptoms in young offenders meeting DSM-5 diagnostic criteria for ADHD. Investigator rated CAARS-O scores is a common outcome measure used in previous treatment trials of ADHD in the community; and measures the same list of 18 symptoms used as the primary outcome in nearly all other studies of Adult ADHD.

- 1. **Secondary endpoints**

Secondary outcomes address important questions about the effects on comorbid symptoms and behavioural impairments that are commonly seen in offenders with ADHD. These are:

• Number of critical incidents (adjudications) from prison records for the 8-week period (in two 4-week periods) from initiation of the trial medication to the at 8- week assessments,

• Ratings of aggressive behaviour by prison staff using the Modified Overt Aggression Scale (MOASP) at 8 weeks

• Behaviour report cards from prison staff (BRC-P) at 8 weeks

• Engagement with educational activities: including: (1) Number of scheduled educational sessions; (2) Reports of disruptive behaviour in education session reported using Classroom behaviour report cards (BRC-E) (completed by education staff only for those people involved in education) at 8 weeks; (3) Ratings of aggressive behaviour by education staff using the Modified Overt Aggression Scale (MOAS) at 8 weeks (for those participating in education only) (MOASE)

• Attitudes towards violence (Maudsley Violence Questionnaire, MVQ) at 8 weeks,

• CORE Outcome Measure (CORE-M) at 8 weeks.

• General psychopathology using the Brief Symptom Inventory (BSI) at 8 weeks,

• Excessive mind wandering measured using the Mind Excessively Wandering Scale (MEWS) at 8 weeks

• Symptoms of emotional dysregulation measured using the WRAADS at 8 weeks,

• Symptoms of emotional dysregulation measured using ARI at 8 weeks,

• Overall health measured using CGI therapeutic score at 8 weeks.

| **Table 1: Primary and secondary outcome measures at 8 weeks** | | | | |
| --- | --- | --- | --- | --- |
| **Concept** | **Instrument (rater)** | **Information used in imputation step of multiple imputation analysis** | **Type of measure** | **Effect size** |
| ADHD symptoms | CAARS-O (investigator rated) | M (B,4,5,8) | Continuous scale | Mean difference |
| Emotional dysregulation | WRAADDS (investigator rated) | M (B,5,8) | Continuous scale | Mean difference |
| Irritability | ARI-S (self-rated) | M (B,5,8) | Continuous scale | Mean difference |
| Spontaneous mind wandering | MEWS (self-rated) | M (B,5,8) | Continuous scale | Mean difference |
| Attitudes towards violence | MVQ (self-rated) | M (B,5,8) | Continuous scale | Mean difference |
| Common psychopathological symptoms | BSI (self-rated) | M (B,5,8) | Continuous scale | Mean difference |
| Global impression of therapeutic effect | CGI-therapeutic (Clinician rated) | M (B,5.8) | Categorical | Mean difference |
| Behavioural problems recorded by prison officers as adjudications in prison records | Critical Incidents (prison records) | Incident rate (B,8) | * Incident rate over 8-week period | Incidence rate ratio |
| Prison office ratings of aggressive behaviour | MOAS-P (prison officer rated) | M (B,8) | Dichotomised (any aggressive event) | Odds ratio |
| Educational staff ratings of aggressive behaviour | MOAS-E (education staff rated) | ** M (B,8) | N/A | N/A |
| Prison officer ratings of behaviour | BRC-P (prison officer rated) | M (B,8) | Proxy count of incidents of inappropriate behaviour | Incidence rate ratio |
| Educational ratings of behaviour | BRC-E (education staff rated) | ** M (B,8) | N/A | N/A |
| Engagement with the educational program | Number education sessions scheduled | ** Incident rate (B,8) | Incident rate over 8-week period | Incidence rate ratio |
| Current psychological distress | CORE-M (self-rated) | M (8) | Continuous | Mean difference |
| **Notes:**   1. N/A refers to too few observations available to warrant formal analysis. 2. M refers to modelling mean 8-week outcomes. 3. The numbers in brackets refer to the assessment time points of measures included in the statistical modelling (B = baseline, 4 = week 4 data, 5 = week 5 data, 8 = week 8 data). 4. ** indicates a measure completed for the subset of participants participating in education and related activities. 5. * For incident rate variables (e.g. number of behavioural problem reports) the baseline data is for 56 days prior to randomisation. For the 8-week outcome the data is from the 56-day period from the start of medication. 6. Incentive and Earned Privileges (IEP) was listed as a secondary outcome in the published protocol, but was removed from the final protocol implemented in December 2018, because IEPs are not recorded in Scottish prisons. | | | | |

1. **STATISTICAL METHODOLOGY**

**2.1 The following approach was used to formally compare the primary and secondary outcomes between the active and placebo arms:**

An intention-to-treat approach was used for all primary and secondary outcomes, that is participants were analysed in the groups to which they were randomised irrespective of adherence with study medication. For each eight-week outcome we estimated the effect of treatment compared to placebo to assess treatment effectiveness.

The primary outcome measure CAARS-O and the secondary outcome measure WRAADDS, MEWS, ARI-S, CGI, BSI, CORE-M and MVQ were continuous variables. Their modelling relied on normal assumptions for error terms and treatment effects were quantified by trial arm differences (and standardised differences).

MOAS-E, MOAS-P and Behaviour report cards (BRC-P, BRC-E) had been expected to follow a normal distribution. Critical Incidents (adjudications) had been expected to follow a Poisson distribution. On review of the distributions of the residuals for a regression model, all of MOAS-P, MOAS-E, BRC-P, BRC-E and critical incidents were noticeably positive skewed and possibly overdispersed or zero inflated.

For critical incidents, which is a count of incidents, the data were positively skewed and zero inflated. However, the outcome was no longer zero inflated with the baseline count of incidents included in the analysis. This was confirmed by checking the residuals. We opted for a negative binomial distribution rather than a Poisson distribution to allow for overdispersion. In addition, we allow for the time in prison as an offset to model the fact that the number of critical incidents are proportional to the time in prison between randomisation and withdrawal or the 8-week assessment timepoint.

Both MOAS-P and BRC-P outcomes were fully observed at baseline. MOAS-P had large zero inflation and too few remaining data points to allow modelling of the distribution (143 of the 200 participants who were included in the study were rated “0” on MOAS-P at eight weeks). We therefore dichotomised this outcome to give a binary variable (1 = participant had any aggressive event, 0 =no aggression), and analysed it is using logistic regression.

BRC-P was approximated by a negative binomial distribution which is appropriate as, although not directly a simple count of incidents, the questionnaire is a weighted proxy count of incidents of inappropriate behaviour. These will be modelled like critical incidents.

Education outcomes were to be analysed only in those persons enrolled in education at baseline. 187 participants were enrolled in some form of education at baseline (94 in OROS-MPH and 93 in placebo) and thus giving a sample size of 187 for this subpopulation. However, only 83 MOAS-E baseline forms were completed, of which, there were only 9 non-zero values. Similarly, only 67 BRC-E forms were completed. We considered an actual sample size of less than half the intended sample size at baseline too small to analyse. Therefore, education outcomes MOASE and BRC-E were not analysed and are only described.

Finally, number of scheduled education sessions at eight weeks was defined as scheduled to attend any of offender training, vocational training, or education sessions. We had intended to use logistic regression to analyse education as a binary variable of whether any education sessions were scheduled or not. At baseline, 187 of 200 participants have any education sessions scheduled. At week eight, 191 of 200 participants have any education scheduled. It was not possible to analyse this using logistic regression given the lack of variability in the data. We therefore analysed this as the underlying count variable “number of any form of education scheduled between baseline and week 8” using a negative binomial distribution to allow for the positive skewness and overdispersion. The proportion of education sessions attended out of those scheduled was also described by arm.

**2.2 Need for multiple imputation**

Formal trial arm comparisons were carried out by multiple imputation (MI), more specifically by using the flexible Multivariate Imputation by Chained equations (MICE) approach⁴’⁵ This was necessary because withdrawal from treatment was found to be predictive of missing primary outcomes (missing CAARS-O at eight weeks). Withdrawal from treatment was defined as; withdrawing completely from the trial, either through choice or through release, transfer or deportation, or withdrawing from treatment only. The association between treatment withdrawal during the trial and missing data in the primary outcome at eight weeks was tested using Fisher’s exact tests. Withdrawal was found to be predictive (p<0.001). Thus, an MI approach was pursued to allow for a missing data generating mechanism that was missing at random (MAR), with the observed variables allowed to drive missingness including withdrawal from treatment.

We empirically assessed whether baseline variables were predictive of missing data using a forward logistic regression modelling approach. The following baseline variables were considered: all co-morbidities as recorded using the MINI checklist, each individual MINI diagnosis, sub-scores of the WRAADDS (emotional over-reactivity, affective lability, temper), MVQ, CGI, Adverse Events Scale (AES), IQ, Childhood trauma questionnaire (CTQ), number of days in prison prior to randomisation, Reactive-Proactive Aggression questionnaire (RPAQ) and the pro-active (RPAQ-P) and re-active (RPAQ-R) subscales, Zanarini Rating Scale for Borderline Personality Disorder (ZAN-BPD) score, Weiss Conduct disorder (WCD), MOAS as recorded by education staff and prison staff (MOASE/P respectively), alcohol or drug usage, ethnicity, their highest level of education, previous treatment for ADHD, and employment. All baseline variables were considered unless one of the categories of the variable occurred too infrequently to expect to observe both missing and observed data (defined as less than 21 observations). Variables were considered to be potentially important, and later considered for inclusion in the imputation model, if they were statistically significant at a liberal 10% test level in a logistic regression of missingness at eight weeks on the variable in question, trial arm and prison (the randomisation stratifier). A single potential predictor variable found. Only employment prior to incarceration was predictive of providing CAARS-O data at eight weeks with those who were in employment, self-employment or education being more likely to provide final outcomes (p-value = 0.075).

MICE was used with regression models for imputation of missing values in continuous variables, with logistic regression models for imputation of missing values in binary variables and with negative binomial models for imputation of missing values in counts.

**2.3 Analysis model**

The analysis models used to estimate treatment effects included the respective outcome variable as the dependent variable and trial arm (trial arm), baseline values of the outcome (if available) and randomisation stratifiers (prison sites) as explanatory variables.

**2.4. Imputation model**

For each outcome variable the imputation model included (i) all the variables of the analysis model, (ii) measures of the outcome variable at baseline and four and/or five weeks if available and (iii) known predictors of missingness (withdrawal from treatment during the trial and employment prior to incarceration). (i) is stipulated by MI theory. (ii) was done to improve the precision of the inferences and also to allow outcome measures at earlier time points to drive drop out at later time points. (iii) accommodates identified predictors of missingness and allowed us to make a more realistic MAR assumption.

Relevant assumptions were checked. Normality and homogeneity assumptions were checked for modelling of CAARS-O, WRAADDS emotional dysregulation sub score, MEWS, ARI-S, CGI, BSI, MVQ, and CORE-M using residual diagnostics. All of these checks were satisfactory. Distribution checks were also done for critical incidents, MOASE/P and BRC-E/P and highlighted that these scores cannot be treated as Poisson or normally distributed (see above).

**2.5. Sensitivity analyses**

Four sets of sensitivity analyses were conducted. Sensitivity analyses were only done on the primary outcome (CAARS-O at eight weeks).

The first sensitivity analysis assessed the assumption that the treatment effect is constant only within defined time windows. That is, only data recorded at week eight visit date plus or minus two days were included in this sensitivity analysis.

The second sensitivity analysis looked at the effect of delayed treatment initiation. Again, those who failed to start treatment within seven days of randomisation were dropped from the analysis of the primary outcome.

The third sensitivity analysis evaluated the impact of IQ on the primary outcome. Those who did not record an IQ greater than 60 on the Wechsler Abbreviated Scale of Intelligence-II (WASI-II) were excluded from this analysis.

The fourth sensitivity analysis assessed efficacy for those who were considered to have complied with treatment. This was defined as taking any trial medication on at least 75% of the days on which it was prescribed. Those who withdrew, or were released, transferred, or deported were excluded.

**2.6. Mechanisms analyses**

**Moderator analyses:** Treatment effect modification by pre-specified putative moderators Childhood trauma questionnaire (CTQ), Zan borderline personality disorder (ZAN-BPD) and Reactive and Proactive sub scores of the Aggression questionnaire (RPAQ) was investigated for the primary outcome CAARS-O at eight weeks. The putative moderators were investigated one at a time. For each moderator, the modelling for CAARS-O at week 8 was extended to include this variable and an interaction between the moderator and the trial arm. To accommodate these variables in the imputation step the “just another variable” approach to multiple imputation with interactions was used; that is both (the fully observed) moderator as well as the product term were simply added to the list of predictors variables available for imputation of missing values. The statistical significance of the interaction term then informs regarding treatment effect modification.

**Mediator analyses:** Treatment effect mediation of behavioural outcomes by 5 week improvements in ADHD symptoms or emotional dysregulation (WRAADDS) was assessed by fitting the following six single mediator models:

Exposure – trial arm; Mediator - CAARS-O hyperactivity subscore; Outcome - BRC-P

Exposure – trial arm; Mediator - CAARS-O inattention subscore; Outcome - BRC-P

Exposure – trial arm; Mediator - CAARS-O WRAADDS emotional dysregulation score; Outcome - BRC-P

Exposure – trial arm; Mediator - CAARS-O hyperactivity subscore; Outcome - Critical Incidents

Exposure – trial arm; Mediator - CAARS-O inattention subscore; Outcome - Critical Incidents

Exposure – trial arm; Mediator - CAARS-O WRAADDS emotional dysregulation score; Outcome - Critical Incidents

Based on complete cases only, causal mediation analyses were performed using the Stata command paramed⁶. In order to base this parametric mediation modelling on linear regression models the respective outcome variables were log-transformed (BRC-P and critical incidents were count outcomes). Baseline values of the mediator and the outcome variable, prison, age, educational level and IQ were included as covariates in the mediator and outcome models because they were hypothesized confounders of the mediator-outcome relationship or a randomisation stratifier (prison). The causal mediation analyses allowed for an interaction between trial arm and the respective mediator. In order to quantify mediation, we report the estimated natural indirect effect (NIE) and the natural direct effect (NDE).

All analyses were carried out in Stata v15.1.

1. **Appendix 2**

**Results from Stastical Analysis Plan report**

- 1. **Baseline characteristics of the sample**

| **Table 2: summaries of clinical baseline variables by trial arm and overall** | | | | | | | |
| --- | --- | --- | --- | --- | --- | --- | --- |
| **Baseline Clinical Characteristics – continuous** | | **OROS-MPH** | | **Placebo** | | **Overall** | |
|  |  | **N** | **Summary** | **N** | **Summary** | **N** | **Summary** |
| CAARS-O - mean (sd) [range 0 to 54] | | 100 | 36.4 (9.8) | 99 | 37.2 (8.7) | 199 | 36.8 (9.2) |
| CAARS-O inattention - mean (sd) [range 0 to 27] | | 101 | 17.9 (5.1) | 99 | 18.5 (4.7) | 200 | 18.2 (4.9) |
| CAARS-O hyperactivity - mean (sd) [range 0 to 27] | | 100 | 18.6 (5.7) | 99 | 18.7 (5.1) | 199 | 18.6 (5.4) |
| RPAQ_P - mean (sd) [range 0 to 24] | | 101 | 6.8 (5.2) | 98 | 7.6 (5.6) | 199 | 7.2 (5.4) |
| RPAQ_R - mean (sd) [range 0 to 22] | | 101 | 14.1 (4.8) | 98 | 14.6 (5.0) | 199 | 14.4 (4.9) |
| RPAQ total - mean (sd) [range 0 to 46] | | 101 | 20.9 (9.2) | 98 | 22.2 (9.7) | 199 | 21.5 (9.4) |
| CTQ - mean (sd) [range 28 to 140] | | 101 | 48.8 (18.8) | 99 | 48.9 (20.7) | 200 | 48.9 (20.7) |
| BMI - mean (sd) | | 101 | 23.7 (3.4) | 99 | 23.7 (3.7) | 200 | 23.7 (3.5) |
| Blood pressure (systolic) - mean (sd) | | 101 | 123.6 (11.2) | 99 | 124.1 (11.9) | 200 | 123.9 (11.5) |
| Blood pressure (diastolic) - mean (sd) | | 101 | 68.2 (9.9) | 99 | 68.1 (9.5) | 200 | 68.2 (9.7) |
| Pulse (beats per minute) - mean (sd) | | 101 | 70.9 (10.7) | 99 | 70.0 (11.8) | 200 | 70.4 (11.2) |
| IQ - mean (sd) [Possible range is 60 or above]* | | 101 | 89.9 (13.5) | 99 | 88.9 (12.4) | 200 | 89.4 (13.0) |
| Weiss CD at baseline [Scale range is 0 to 45] | | 101 | 17.9 (7.7) | 99 | 18.7 (7.8) | 200 | 18.3 (7.7) |
| CGI - Severity of illness - mean (sd)  [Range from 1 – Normal, not at all ill through 4 - Moderately ill to 7 - Among the most extremely ill patients] | | 101 | 4.0 (1.0) | 99 | 3.9 (1.1) | 200 | 3.9 (1.0) |
| BRC-P at baseline - min/median/max | | 101 | 6/8/24 | 99 | 6/8/21 | 200 | 6/8/24 |
| BRC-E at baseline - min/median/max | | 31 | 13.00/  18.55/  41.45 | 36 | 13.00/  17.45/  40.00 | 67 | 13.00/  18.55/  41.45 |
| MOASP - MOAS recorded by Prison officer - mean (sd)  min/ median / max  [Scale range is from 0 to 40] | | 101 | 0/0/15 | 99 | 0/0/19 | 200 | 0/0/19 |
| MOASE - MOAS recorded by Education staff - mean (sd)  min/ median / max  [Scale range is from 0 to 40] | | 38 | 0/0/12 | 45 | 0/0/10 | 83 | 0/0/12 |
| MVQ - mean (sd)  [Scale range is 0 to 75] | | 101 | 33.2 (9.4) | 99 | 34.6 (9.9) | 200 | 33.9 (9.6) |
| MEWS - mean (sd)  [Scale range is 0 to 36] | | 101 | 25.7 (6.7) | 99 | 26.8 (6.2) | 200 | 26.3 (6.5) |
| WRAADDS emotional dysregulation – mean (sd)  [Scale range is 0 to 30] | | 101 | 17.5 (5.7) | 99 | 18.1 (5.6) | 200 | 17.8 (5.7) |
| WRAADDS Temper - mean (sd)  [Scale range is 0 to 9] | | 101 | 4.7 (2.5) | 99 | 5.2 (2.3) | 200 | 4.9 (2.4) |
| WRAADDS Lability - mean (sd)  [Scale range is 0 to 12] | | 101 | 8.0 (2.3) | 99 | 8.1 (2.2) | 200 | 8.0 (2.2) |
| WRAADDS Over-reactivity - mean (sd)  [Scale range is 0 to 9] | | 101 | 4.8 (2.2) | 99 | 4.8 (2.3) | 200 | 4.8 (2.2) |
| ARI-S - mean (sd) [Scale range is 0 to 14] | | 101 | 9.3 (3.5) | 99 | 9.3 (3.7) | 200 | 9.3 (3.6) |
| BSI - mean (sd) [Scale range is 0 to 212] | | 101 | 52.5 (32.5) | 99 | 52.9 (35.9) | 200 | 52.7 (34.2) |
| ZAN-BPD - mean (sd)  [Scale range is 0 to 36] | | 101 | 6.9 (5.1) | 99 | 6.3 (4.2) | 200 | 6.6 (4.6) |
| CORE-M – mean (sd)  [Scale range is 0 to 136] | | 101 | 43.5 (13.9) | 99 | 44.8 (15.3) | 200 | 44.2 (14.6) |
| Critical Incidents at baseline - min/median/max | | 101 | 0/0/6 | 99 | 0/0/10 | 200 | 0/0/10 |
| Number of days in prison (in 56 days before randomisation) - min/median/max | | 101 | 24/56/56 | 99 | 27/56/56 | 200 | 24/56/56 |
| Number of Education sessions (any type) scheduled at baseline - min/median/max | | 101 | 0/21/123 | 99 | 0/25/126 | 200 | 0/23/126 |
| Number of Education sessions (any type) attended at baseline - min/median/max | |  | 0/15/112 |  | 0/19/126 |  | 0/17/126 |
| Proportion of schedule education sessions (any type) that were attended at baseline (in those scheduled for at least 1 education session) - mean (sd) | |  | 0.78 (0.31) |  | 0.82 (0.26) |  | 0.81 (0.29) |
| **Baseline Clinical Characteristics from MINI - binary (No/Yes)** | | **N** | **Summary** | **N** | **Summary** | **N** | **Summary** |
| Antisocial Personality Disorder - n (%) | No | 101 | 29 (28.7) | 99 | 22 (22.2) | 200 | 51 (25.5) |
|  | Yes |  | 72 (71.3) |  | 77 (77.8) |  | 149 (74.5) |
| Mood (major depression, suicidality, manic, hypomanic) - n (%) | No | 101 | 71 (70.3) | 99 | 66 (66.7) | 200 | 137 (68.5) |
|  | Yes |  | 30 (29.7) |  | 33 (33.3) |  | 63 (31.5) |
| Anxiety (panic, agoraphobia, social anxiety, obsessive-compulsive disorder) – n (%) | No | 101 | 82 (81.2) | 99 | 80 (80.8) | 200 | 162 (81.0) |
|  | Yes |  | 19 (18.8) |  | 19 (19.2) |  | 38 (19.0) |
| Psychotic Disorder - n (%) | No | 101 | 101 (100.0) | 99 | 99 (100.0) | 200 | 200 (100.0) |
| **Mini checklist (MC) sub-categories**  **[Scale range is 0 – not present to 10 – extreme]** | | | | | | | |
| Depression - min/med/max | | 101 | 0/1/7 | 99 | 0/1/8 | 200 | 0/1/8 |
| Anger - min/med/max | | 101 | 0/4/9 | 99 | 0/5/9 | 200 | 0/4/9 |
| Mania - min/med/max | | 101 | 0/0/6 | 99 | 0/0/3 | 200 | 0/0/6 |
| Anxiety - min/med/max | | 101 | 0/1/8 | 99 | 0/1/8 | 200 | 0/1/8 |
| Physical symptoms - min/med/max | | 101 | 0/0/6 | 99 | 0/0/5 | 200 | 0/0/6 |
| Suicidal thoughts - min/med/max | | 101 | 0/0/3 | 99 | 0/0/3 | 200 | 0/0/3 |
| Psychosis - min/med/max | | 101 | 0/0/1 | 99 | 0/0/3 | 200 | 0/0/3 |
| Sleep problems - min/med/max | | 101 | 0/4/9 | 99 | 0/4/10 | 200 | 0/4/10 |
| Memory problems - min/med/max | | 101 | 0/3/7 | 99 | 0/3/7 | 200 | 0/3/7 |
| Repetitive thoughts/behaviours - min/med/max | | 101 | 0/0/7 | 99 | 0/0/7 | 200 | 0/0/7 |
| Dissociation - min/med/max | | 101 | 0/0/0 | 99 | 0/0/5 | 200 | 0/0/5 |
| Personality functioning – min/med/max | | 101 | 0/2/9 | 99 | 0/2/8 | 200 | 0/2/9 |

*WASI-II (IQ) score less than 60 in one individual (eligible on clinical grounds)

- 1. Figure 1: Mean weekly CAARS-O score throughout the trial for OROS-methylphenidate and placebo arms.

- 1. **Responder rates for primary outcome**

| **Table 3: Responders by trial arm** | | | | |
| --- | --- | --- | --- | --- |
| **Clinical Characteristics - outcomes – continuous** | | **OROS-MPH**  **(number recorded =89)** | **Placebo**  **(number recorded = 94)** | **Overall** |
| CAARS-O reduction (as a % of baseline) – mean (SD) |  | 21.24 (33.81) | 20.12 (29.73) | 20.66 (31.69) |
| CAARS –O responders at 8 weeks - n (%) | Non-responders | 46 (51.69) | 49 (52.13) | 95 (51.91) |
|  | Responders | 43 (48.31) | 45 (47.87) | 88 (48.09) |
| CAARS-O Inattention reduction (as a % of baseline) – mean (SD) |  | 26.40 (36.89) | 22.12 (35.64) | 24.22 (36.12) |
| CAARS-O Inattention responders at 8 weeks - n (%) | Non-responders | 40 (44.44) | 43 (45.74) | 83 (45.11) |
|  | Responders | 50 (55.56) | 51 (54.26) | 101 (54.89) |
| CAARS-O Hyperactivity reduction (as a % of baseline) – mean (SD) |  | 11.67 (59.32) | 16.33 (36.44) | 14.08 (48.78) |
| CAARS-O Hyperactivity responders at 8 weeks - n (%) | Non-responders | 49 (55.68) | 50 (53.19) | 99 (54.40) |
|  | Responders | 39 (44.32) | 44 (46.81) | 83 (45.60) |

- 1. **Sensitivity Analyses:**

Four sets of sensitivity analyses were conducted to check whether the results on the primary outcome were sensitive to collection of outcomes within the specified window, starting treatment within the specified time, sufficiently high IQ confirmed by a standardised test, or sufficient level of compliance with the treatment. In each case, the primary analysis, estimating the difference between trial arms in CAARS-O at 8 weeks, was repeated. The primary analysis was robust to all assumptions tested in these sensitivity analyses.

**Sensitivity analysis 1: Outcome window**

As per protocol the primary outcome time was at 8 weeks or 56 days, and not more than 2 days before or after the expected date. Six participants had the primary outcome completed at more than 58 days and 18 people before 54 days. Of 176 participants who completed final outcomes on time, the estimated difference was 0.729 (95% CI -2.353 to 3.811, p=0.641) which does not differ appreciably from the primary analysis.

**Sensitivity analysis 2: Acceptable treatment start time**

Participants, once randomised, should have been prescribed a first dose and initiated treatment within no more than 7 days of randomisation. 14 of the 200 did not start treatment within the defined time. Of 186 participants who started treatment on time, the estimated difference was 0.652 (95% CI -2.474 to 3.778, p=0.681) which does not differ appreciably from the primary analysis.

**Sensitivity analysis 3: IQ**

ADHD symptoms can make IQ tests less reliable. Clinical judgement was used to assess IQ in addition to the use of the Wechsler Abbreviated Scale of Intelligence-II (WASI-II) IQ test. One participant was measured as having an IQ below 60 on the test. This sensitivity analysis confirms the result is valid if the sample is restricted to those people who met the eligibility criterion relating to IQ test. The estimated difference was 0.512 (95% CI -2.507 to 3.530, p=0.738.

**Sensitivity analysis 4: Compliance with treatment**

83 participants complied with trial medication according to our definition of compliance of taking some or all of their prescribed trial medication on at least 75% of the days for which it was prescribed. 34 participants complied in the OROS-MPH arm compared to 49 in the placebo arm. The estimated trial arm difference in the compliers (-0.29) was a small difference in the opposite direction (a greater effect in the placebo arm) but was not statistically significant (95% CI -5.09, 4.52, p=0.91).

- 1. Mediator analyses

| **Table 4: NDE, NIE and MTE for potential mediators (complete case analyses)** | | | |
| --- | --- | --- | --- |
| **Mediator (M) and outcome variable (O)** | **NDE(95% CI)** | **NIE (95% CI)** | **TE**  **(95% CI)** |
| M: CAARS-O hyperactivity sub score |  |  |  |
| O: BRC-P | -0.06 (-0.16, 0.04) | 0.00 (-0.01, 0.01) | -0.06 (-0.16, 0.04) |
| O: Critical Incidents | -0.66 (-1.36, 0.05) | -0.01 (-0.008, 0.06) | -0.67 (-1.37, 0.03) |
| M CAARS-O inattention sub score |  |  |  |
| O: BRC-P | -0.06 (-0.15, 0.05) | 0.00 (-0.01, 0.03) | -0.06 (-0.15, 0.05) |
| O: Critical Incidents | -0.69 (-1.41, 0. 02) | 0.00 (-0.05, 0.05) | -0.69 (-1.40, 0.02) |
| M: WRAADDS emotional dysregulation score |  |  |  |
| O: BRC-P | -0.06 (-0.16, 0.04) | 0.00 (-0.01, 0.00) | -0.06 (-0.16, 0.04) |
| O: Critical Incidents | -0.67 (-1.37, 0.03) | -0.03 (-0.06, 0.12) | -0.64 (-1.34, 0.06) |

*The estimates of the total effects can differ between models due to varying completeness of recording of different mediators.

- 1. Moderator analyses

| **Table 5: Formal assessment of moderation effects** | | | |
| --- | --- | --- | --- |
| **Baseline variable** | **Estimated interaction effect** | **95% CI** | **t-test, degrees of freedom, p-value** |
| Interaction of ZAN-BPD with trial arm | 0.56 | (-0.09, 1.20) | 1.70, 181, 0.090 |
| Interaction of CTQ with trial arm | 0.10 | (-0.06, 0.25) | 1.25, 171, 0.214 |
| Interaction of RPAQ-R with trial arm | 0.23 | (-0.37, 0.83) | 0.75, 176, 0.452 |
| Interaction of RPAQ-P with trial arm | 0.28 | (-0.26, 0.82) | 1.02, 180, 0.113 |

- 1. **Adverse events**

| **Table 6: Adverse events by body system code by trial arm** | | | | | | | |
| --- | --- | --- | --- | --- | --- | --- | --- |
| **SOC category** | **HLGT category** | **OROS-MPH**  **n of participants/%** | **OROS-MPH**  **n of events/%** | **Placebo**  **n of participants /%** | **Placebo**  **n of events/%** | **Total**  **n of participants /%** | **Total**  **n of events/%** |
| Blood and lymphatic system disorders | Blood and lymphatic system disorders | 1 / 0.54 | 1 / 0.45 | 0 / 0.0 | 0 / 0.0 | 1 / 0.30 | 1/ 0.24 |
| Cardiac disorders | Cardiac signs and symptoms | 4 / 2.17 | 4/ 1.79 | 1 / 0.66 | 1/ 0.52 | 5 / 1.49 | 5/ 1.20 |
| Ear and labyrinth disorders | External ear disorder | 3 / 1.63 | 3/ 1.35 | 0 / 0.0 | 0 / 0.0 | 3 / 0.89 | 3/ 0.72 |
| Endocrine | Endocrine | 0 / 0.0 | 0/0.0 | 1 / 0.66 | 1/ 0.52 | 1 / 0.30 | 1/ 0.24 |
| Eye disorders | Eye disorders not otherwise specified | 1 / 0.54 | 1/ 0.45 | 0 / 0.0 | 0 / 0.0 | 1 / 0.30 | 1/ 0.24 |
|  | Ocular infection infestation, irritations and inflammation | 0 / 0.0 | 0 / 0.0 | 2 / 1.32 | 2/ 1.04 | 2 / 0.60 | 2/ 0.48 |
| Gastrointestinal disorders | Dental and gum disorders | 22 / 11.96 | 35/ 15.70 | 15 / 9.87 | 20/ 10.36 | 37 / 11.01 | 55/ 13.22 |
|  | Gastrointestinal NOS | 0 / 0.0 | 0/0.0 | 2 / 1.32 | 2/ 1.04 | 2 / 0.60 | 2/ 0.48 |
|  | Gastrointestinal motility and defaecation disorders | 11 / 5.98 | 11/ 4.93 | 11 / 7.24 | 12/ 6.22 | 22 / 6.55 | 23/ 5.53 |
| General disorders | General disorders NOS | 12 / 6.52 | 13/ 5.83 | 7 / 4.61 | 7/ 3.63 | 19 / 5.65 | 20/ 4.81 |
| Immune system disorders | Allergic conditions | 5 / 2.72 | 5/ 2.24 | 9 / 5.92 | 13/ 6.74 | 14 / 4.17 | 18/ 4.33 |
| Infections and infestations | Infections and infestations | 6 / 3.26 | 7/ 3.14 | 12 / 7.89 | 14/ 7.25 | 18 / 5.36 | 21/ 5.05 |
| Injury, poisoning and procedural complications | Bone and joint injuries | 1 / 0.54 | 1/ 0.45 | 5 / 3.29 | 9/ 4.66 | 6 / 1.79 | 10/ 2.40 |
|  | Soft tissue injury | 11 / 5.98 | 14/ 6.28 | 8 / 5.26 | 8/ 4.15 | 19 / 5.65 | 22/ 5.29 |
| Metabolism and nutrition disorder | Appetite and general nutritional disorders | 13 / 7.07 | 14/ 6.28 | 2 / 1.32 | 2/ 1.04 | 15 / 4.46 | 16/ 3.85 |
|  | Vitamin related disorders | 1 / 0.54 | 1/ 0.45 | 1 / 0.66 | 1/ 0.52 | 2 / 0.60 | 2/ 0.48 |
| Musculoskeletal and connective tissue disorders | Musculoskeletal disorders | 15 / 8.15 | 17/ 7.62 | 13 / 8.55 | 16/ 8.29 | 28 / 8.33 | 33/ 7.93 |
| Neoplasms benign, malignant and unspecified | Benign neoplasm | 1 / 0.54 | 1/ 0.45 | 2 / 1.32 | 2/ 1.04 | 3 / 0.89 | 3/ 0.72 |
| Nervous system disorders | Dizziness | 6 / 3.26 | 6/ 2.69 | 0 / 0.0 | 0 / 0.0 | 6 / 1.79 | 6/ 1.44 |
|  | Headache | 17 / 9.24 | 27/ 12.11 | 14 / 9.21 | 26/ 13.47 | 31/ 9.23 | 53/ 12.74 |
|  | Seizures | 1 / 0.54 | 1/ 0.45 | 0 / 0.0 | 0 / 0.0 | 1 / 0.30 | 1/ 0.24 |
| Psychiatric disorder | Conditions associated with drug abuse | 4 / 2.17 | 7/ 3.14 | 9 / 5.92 | 13/ 6.74 | 13 / 3.87 | 20/ 4.81 |
|  | Depressed mood disorders and disturbances | 12 / 6.52 | 13/ 5.83 | 4 / 2.63 | 6/ 3.11 | 16 / 4.76 | 19/ 4.57 |
|  | Psychiatric and behavioural symptoms | 5 / 2.72 | 5/ 2.24 | 5 / 3.29 | 5/ 2.59 | 10 / 2.98 | 10/ 2.40 |
|  | Sleep disorders and disturbances | 11 / 5.98 | 11/ 4.93 | 7 / 4.61 | 7/ 3.63 | 18 / 5.36 | 18/ 4.33 |
|  | Somatic symptom and related disorders | 3 / 1.63 | 3 / 1.35 | 0 / 0.0 | 0 / 0.0 | 3 / 0.89 | 3/ 0.72 |
| Renal and urinary disorder | Urinary problem NOS | 1 / 0.54 | 1 / 0.45 | 0 / 0.0 | 0 / 0.0 | 1 / 0.30 | 1 / 0.24 |
| Respirator, thoracic and mediastinal disorders | Respiratory, thoracic and mediastinal disorders | 1 / 0.54 | 1 / 0.45 | 0 / 0.0 | 0 / 0.0 | 1 / 0.30 | 1 / 0.24 |
| Skin and subcutaneaous disorder | Epidermal and dermal conditions | 16 / 8.70 | 20 / 8.97 | 22 / 14.47 | 26 / 13.47 | 38 / 11.31 | 46 / 11.06 |

| **Table 7: Summaries of the Adverse Events Scale by trial arm and overall** | | | | | | |
| --- | --- | --- | --- | --- | --- | --- |
| **Adverse Events Scale (AES)**  **[Scale range is 0 to 69]** | **OROS-MPH** | | **Placebo** | | **Overall** | |
|  | **Number**  **recorded** | **Mean (sd)** | **Number**  **recorded** | **Mean (sd)** | **Number**  **recorded** | **Mean (sd)** |
| Baseline | 101 | 10.29 (7.51) | 99 | 9.81 (7.27) | 200 | 10.05 (7.38) |
| Week 8 | 89 | 7.42 (7.46) | 93 | 5.52 (5.42) | 182 | 6.45 (6.55) |

| **Table 8: Adverse Events Scale detail – numbers and percentage with the symptom – baseline** | | | |
| --- | --- | --- | --- |
| **Adverse Events Scale (AES) items** | **OROS-MPH**  **(N=101)**  **Number (%)** | **Placebo**  **(N=99)**  **Number (%)** | **Overall**  **(N=200)**  **Number (%)** |
| Headache | 16 (15.84) | 15 (15.15) | 31 (15.50) |
| Dryness of the skin | 12 (11.88) | 17 (17.17) | 29 (14.50) |
| Dryness of the eyes | 1 (0.99) | 1 (1.01) | 2 (1.00) |
| Dryness of the mouth | 5 (4.95) | 7 (7.07) | 12 (6.00) |
| Thirst | 16 (15.84) | 16 (16.16) | 32 (16.00) |
| Sore throat | 5 (4.95) | 1 (1.01) | 6 (3.00) |
| Dizziness | 4 (3.96) | 1 (1.01) | 5 (2.50) |
| Nausea | 0 (0.00) | 2 (2.02) | 2 (1.00) |
| Stomach aches | 2 (1.98) | 3 (3.03) | 5 (2.50) |
| Vomiting | 0 (0.00) | 0 (0.00) | 0 (0.00) |
| Sweating | 9 (8.91) | 5 (5.05) | 14 (7.00) |
| Appetite reduction | 6 (5.94) | 8 (8.08) | 14 (7.00) |
| Diarrhea | 2 (1.98) | 1 (1.01) | 3 (1.50) |
| Frequent urination | 4 (3.96) | 7 (7.07) | 11 (5.50) |
| Tics | 3 (2.97) | 6 (6.06) | 9 (4.50) |
| Sleep difficulties | 52 (51.49) | 50 (50.51) | 102 (51.00) |
| Mood instability | 32 (31.68) | 45 (45.45) | 77 (38.50) |
| Irritability | 38 (37.62) | 37 (37.37) | 75 (37.50) |
| Agitation/Excitability | 38 (37.62) | 39 (39.39) | 77 (38.50) |
| Sadness | 9 (8.91) | 16 (16.16) | 25 (12.50) |
| Heart palpitations | 4 (3.96) | 4 (4.04) | 8 (4.00) |
| Sexual dysfunction | 2 (1.98) | 0 (0.00) | 2 (1.00) |

| **Table 9: Adverse Events Scale detail – numbers and percentage with the symptom – across the trial period** | | | |
| --- | --- | --- | --- |
| **Adverse Events Scale (AES) items** | **OROS-MPH**  **(N=101)**  **Number (%)** | **Placebo**  **(N=99)**  **Number (%)** | **Overall**  **(N=200)**  **Number (%)** |
| Headache | 18 (17.82) | 10 (10.10) | 28 (14.00) |
| Dryness of the skin | 14 (13.86) | 18 (18.18) | 32 (16.00) |
| Dryness of the eyes | 2 (1.98) | 4 (4.04) | 6 (3.00) |
| Dryness of the mouth | 20 (19.80) | 10 (10.10) | 30 (15.00) |
| Thirst | 22 (21.78) | 18 (18.18) | 40 (20.00) |
| Sore throat | 8 (7.92) | 6 (6.06) | 14 (7.00) |
| Dizziness | 6 (5.94) | 3 (3.03) | 9 (4.50) |
| Nausea | 8 (7.92) | 3 (3.03) | 11 (5.50) |
| Stomach aches | 7 (6.93) | 3 (3.03) | 10 (5.00) |
| Vomiting | 1 (0.99) | 1 (1.01) | 2 (1.00) |
| Sweating | 20 (19.80) | 8 (8.08) | 28 (14.00) |
| Appetite reduction | 35 (34.65) | 19 (19.19) | 54 (27.00) |
| Diarrhea | 6 (5.94) | 1 (1.01) | 7 (3.50) |
| Frequent urination | 14 (13.86) | 11 (11.11) | 25 (12.50) |
| Tics | 3 (2.97) | 6 (6.06) | 9 (4.50) |
| Sleep difficulties | 56 (55.45) | 50 (50.51) | 106 (53.00) |
| Mood instability | 40 (39.60) | 46 (46.46) | 86 (43.00) |
| Irritability | 49 (48.51) | 49 (49.49) | 98 (49.00) |
| Agitation/Excitability | 42 (41.58) | 43 (43.43) | 85 (42.50) |
| Sadness | 17 (16.83) | 19 (19.19) | 36 (18.00) |
| Heart palpitations | 6 (5.94) | 3 (3.03) | 9 (4.50) |
| Sexual dysfunction | 2 (1.98) | 0 (0.00) | 2 (1.00) |

- 1. **Vital signs**

| **Table 10: Summaries of vital signs by trial arm and overall** | | | | | | | | |  |
| --- | --- | --- | --- | --- | --- | --- | --- | --- | --- |
| **Vital signs** | **OROS-MPH** | | **Placebo** |  | **Overall** | |  | |  |
|  | **N** | **Summary**  **Mean (sd)** | **N** | **Summary**  **Mean (sd)** | **N** | | **Summary**  **Mean (sd)** | |  |
| BMI at week 0 | 101 | 23.7 (3.4) | 99 | 23.7 (3.7) | | 200 | | 23.7 (3.5) | |
| BMI at week 5 | 88 | 23.4 (3.5) | 88 | 24.2 (3.8) | | 176 | | 23.8 (3.7) | |
| BMI at week 8 | 86 | 23.6 (3.4) | 87 | 24.2 (3.8) | | 173 | | 23.9 (3.6) | |
| Blood pressure (systolic) at week 1 | 98 | 124.1 (10.3) | 98 | 125.2 (11.9) | | 196 | | 124.7 (11.0) | |
| Blood pressure (diastolic) at week 1 | 98 | 71.8 (9.7) | 98 | 70.8 (10.1) | | 196 | | 71.3 (9.9) | |
| Pulse (beats per minute) at week 1 | 98 | 76.5 (11.4) | 98 | 72.1 (10.4) | | 196 | | 74.3 (11.1) | |
| Blood pressure (systolic) at week 2 | 92 | 124.5 (9.4) | 98 | 124.6 (12.1) | | 190 | | 124.5 (10.8) | |
| Blood pressure (diastolic) at week 2 | 92 | 70.9 (9.1) | 98 | 70.9 (10.0) | | 190 | | 70.9 (8.5) | |
| Pulse (beats per minute) at week 2 | 92 | 75.8 (11.7) | 98 | 74.4 (13.6) | | 190 | | 75.1 (12.7) | |
| Blood pressure (systolic) at week 3 | 93 | 124.1 (12.3) | 95 | 122.5 (10.2) | | 188 | | 123.3 (11.3) | |
| Blood pressure (diastolic) at week 3 | 93 | 72.0 (10.0) | 95 | 69.8 (9.4) | | 188 | | 70.9 (9.7) | |
| Pulse (beats per minute) at week 3 | 93 | 75.7 (12.1) | 95 | 71.5 (10.8) | | 188 | | 73.6 (11.6) | |
| Blood pressure (systolic) at week 4 | 92 | 124.1 (13.3) | 96 | 125.2 (14.6) | | 188 | | 124.6 (13.9) | |
| Blood pressure (diastolic) at week 4 | 92 | 72.5 (11.2) | 96 | 69.6 (9.0) | | 188 | | 71.0 (10.2) | |
| Pulse (beats per minute) at week 4 | 92 | 75.9 (12.7) | 96 | 73.5 (11.5) | | 188 | | 74.7 (12.1) | |
| Blood pressure (systolic) at week 5 | 91 | 124.4 (11.5) | 93 | 124.7 (12.0) | | 184 | | 124.6 (11.8) | |
| Blood pressure (diastolic) at week 5 | 91 | 70.7 (10.3) | 93 | 69.9 (10.2) | | 184 | | 70.3 (10.2) | |
| Pulse (beats per minute) at week 5 | 91 | 75.0 (12.5) | 93 | 72.9 (10.6) | | 184 | | 73.9 (11.6) | |
| Blood pressure (systolic) at week 8 | 89 | 125.0 (12.5) | 93 | 125.5 (14.0) | | 182 | | 125.2 (13.2) | |
| Blood pressure (diastolic) at week 8 | 89 | 70.9 (11.6) | 93 | 70.6 (9.3) | | 182 | | 70.8 (10.4) | |
| Pulse (beats per minute) at week 8 | 89 | 74.8 (11.2) | 93 | 71.9 (11.4) | | 182 | | - 1. (11.3) | |

- 1. **PRESCRIBED DOSE AND ADHERENCE TO MEDICATION**

| **Table 11: Summaries of daily tablets prescribed by week, trial arm and overall** | | | |
| --- | --- | --- | --- |
| **Week number (number of participants continuing treatment)** | **OROS-MPH**  **(N=101)**  **Mean (s.d.)** | **Placebo**  **(N=99)**  **Mean (s.d.)** | **Overall**  **(N=200)**  **Mean (s.d.)** |
| 1 (N=200) | 1.00 (0.00) | 1.00 (0.00) | 1.00 (0.00) |
| 2 (N=197) | 1.66 (0.50) | 1.71 (0.46) | 1.68 (0.48) |
| 3 (N=191) | 2.20 (0.80) | 2.44 (0.67) | 2.32 (0.75) |
| 4 (N=183) | 2.71 (1.08) | 3.03 (1.00) | 2.87 (1.05) |
| 5 (N=177) | 2.99 (1.21) | 3.41 (0.84) | 3.21 (1.05) |

| **Table 12: Summaries of daily tablets taken by week, trial arm and overall** | | | | | | |
| --- | --- | --- | --- | --- | --- | --- |
| **Week** | **OROS-MPH**  **Mean (s.d.)** | **OROS-MPH**  **percentage of prescribed** | **Placebo**  **Mean (s.d.)** | **Placebo percentage of prescribed** | **Overall**  **Mean (s.d.)** | **Overall percentage of prescribed** |
| 1 (N=200) | 0.76 (0.32) | 76.0 | 0.86 (0.23) | 86.0 | 0.81 (0.28) | 81.0 |
| 2 (N=197) | 1.23 (0.69) | 74.1 | 1.35 (0.60) | 78.9 | 1.29 (0.65) | 76.8 |
| 3 (N=191) | 1.49 (0.98) | 67.7 | 1.89 (0.88) | 77.5 | 1.69 (0.95) | 72,8 |
| 4 (N=183) | 1.55 (1.22) | 57.2 | 2.22 (1.20) | 90.9 | 1.88 (1.25) | 65.5 |
| 5 (N=177) | 1.60 (1.38) | 53.5 | 2.40 (1.27) | 70.4 | 2.00 (1.38) | 62.3 |
| 6 (N=171) | 1.57 (1.38) | 52.5 | 2.33 (1.32) | 68.3 | 1.95 (1.40) | 60.7 |
| 7 (N=171) | 1.59 (1.38) | 53.2 | 2.25 (1.43) | 66.0 | 1.92 (1.44) | 59.8 |
| 8 (N=158) | 1.41 (1.34) | 47.2 | 1.99 (1.41) | 58.4 | 1.70 (1.40) | 53.0 |

Note that tablets were recorded in the database as prescribed at weeks 1,2,3,4,5. The number prescribed was assumed to be constant from week 5 onwards. The number of participants shown is the number of participants who were recorded as being actively prescribed up to and including week 5 and at week 8. The status of participants was not recorded at week 6 and 7. Participants who withdrew from treatment according to their recorded status or the trial are not counted in the number, neither are those given a zero prescription.

**Figure 2: Weekly mean number of tablets taken by trial arm**

- 1. **CONCOMITANT MEDICATION**

| **Table 13: Concomitant medication at baseline** | | | |
| --- | --- | --- | --- |
| Class of Drug | OROS-MPH N (number of participants) % | Placebo N (number of participants) % | Overall N (number of participants) % |
| Antidepressant | 13 (56.5) | 10 (43.5) | 23 (100.0) |
| Antihistamine | 2 (66.7) | 1 (33.3) | 3 (100.0) |
| Antipsychotic | 2 (33.3) | 4 (66.7) | 6 (100.0) |
| Methadone | 0 (0.0) | 1 (100.0) | 1 (100.0) |
| Nicotine | 5 (55.6) | 4 (44.4) | 9 (100.0) |
| Non-psychotropic | 24 (40.7) | 35 (59.3) | 59 (100.0) |
| Propanolol | 4 (80.0) | 1 (20.0) | 5 (100.0) |
| Sodium Valporate | 1 (100.0) | 0 (0.0) | 1 (100.0) |
| Total | 51 (47.7) | 56 (52.3) | 107 (100.0) |

| **Table 13: Concomitant medications during the trial** | | | |
| --- | --- | --- | --- |
| Class of Drug | OROS-MPH N (number of participants) % | Placebo N (number of participants) % | Overall N (number of participants) % |
| Antidepressant | 1 (33.3) | 2 (66.7) | 3 (100.0) |
| Antihistamine | 6 (50.0) | 6 (50.0) | 12 (100.0) |
| Antipsychotic | 1 (100.0) | 0 (0.0) | 1 (100.0) |
| Concerta XL | 1 (100.0) | 0 (0.0) | 1 (100.0) |
| Herbal sedative | 1 (100.0) | 0 (0.0) | 1 (100.0) |
| Melatonin | 1 (50.0) | 1 (50.0) | 2 (100.0) |
| Nicotine | 6 (60.0) | 4 (40.0) | 10 (100.0) |
| Non-psychotropic | 29 (48.3) | 31 (51.7) | 60 (100.0) |
| Propanolol | 0 (0.0) | 1 (100.0) | 1 (100.0) |
| Sodium Valporate | 1 (100.0) | 0 (0.0) | 1 (100.0) |
| Total | 47 (51.1) | 45 (48.9) | 92 (100.0) |

- 1. **Illicit drug use**

| **Table 15: Illicit drug use prior to incarceration** | | | |
| --- | --- | --- | --- |
| Class of drug | OROS-MPH N (Number of people taking drug) (% of drugs taken) | Placebo N (Number of people taking drug) (% of drugs taken) | Overall N (Number of people taking drug) (% of drugs taken) |
| Cannabis | 97 (32.23) | 93 (32.52) | 190 (32.37) |
| Cocaine | 53 (17.61) | 48 (16.78) | 101 (17.21) |
| Methamphetamine | 28 (9.30) | 21 (7.34) | 49 (8.35) |
| Inhalants | 4 (1.33) | 5 (1.75) | 9 (1.53) |
| Sedatives or sleeping pills | 22 (7.31) | 21 (7.34) | 43 (7.33) |
| Hallucinogens | 38 (12.62) | 28 (9.79) | 66 (11.24) |
| Street opioids | 5 (1.66) | 4 (1.40) | 9 (1.53) |
| Prescription opioids | 10 (3.32) | 16 (5.59) | 26 (4.43) |
| Spice | 12 (3.99) | 21 (7.34) | 33 (5.62) |
| Other | 30 (9.97) | 25 (8.74) | 55 (9.37) |
| None | 2 (0.66) | 4 (1.40) | 6 (1.02) |

**APPENDIX 3:**

**ADDITIONAL INVESTIGATIONS**

1. **Introduction**

The findings from the primary trial analysis according to our pre-specified Statistical Analysis Plan (SAP) provide no evidence for difference in effect between the placebo and treatment arms. Indeed, across all primary and secondary outcome variables outcomes were remarkably similar in the trial arms at 8 weeks and do not indicate any advantage of OROS-MPH over placebo, even at trend level. Furthermore, findings from the per protocol analyses as laid out in the SAP did not provide any evidence for efficacy of the OROS-MPH intervention either.

As outlined in the justification for this study, methylphenidate has been investigated in adults with ADHD in community ADHD clinics in previous studies and found to have an average standardised between-group effect of around SMD = 0.51. A recent comprehensive network meta-analysis estimated an effect size from randomised controlled trials of MPH in reduction of ADHD symptoms in adults with an SMD of 0.49 (95% CI: 0.35 to 0.64). One of the key rationales for this study was to investigate whether OROS-MPH had a different effect in a young adult male prison population when compared to the previous studies. It was proposed that the symptoms of inattention and hyperactivity/impulsivity could potentially have a different meaning in this population reflecting different comorbid mental health or neurodevelopmental disorders. It was further proposed that high levels of drug and alcohol use among offenders might modify the previously reported effects. While a change in the effect size compared to previous studies was envisaged, the absence of even a small effect was unexpected (the estimated between arm standardised effect is < 0.1).

While there appeared to be no between-group effects, there was a change over the observation period indicating an apparent improvement in symptoms in both arms. CAARS-O scores dropped from 36.4 to 28.0 (8.4 points) in the OROS-MPH arm of the trial and from 37.2 to 29.3 (7.9 points) in the placebo arm (Table 9). This is not unexpected since significant change has been observed in placebo arms of most randomised controlled trials for MPH in ADHD. However, we saw much greater change in symptoms in the pilot study for this project at HMP YOI Isis, which included 121 participants in a single unblinded group receiving OROS-MPH ^29^. The pilot study found the mean change over time for CAARS-O to be 22.6 (37.6 to 15.0) using the primary outcome at week 12 and last observation carried forward approach to deal with missing observations. For the week 8 secondary outcome data in the pilot using the LOCF approach, the difference was even greater: 29.3 (from 37.6 to 8.31). This appears to be far larger than the change observed in either of the two treatment arms of the current trial.

For these reasons we completed a set of further analyses to explore possible explanations for the unexpected findings in this study. These further analyses were proposed after database lock and review of the findings from the primary trial analyses according to the SAP. They clearly are post-hoc analyses and should be regarded as entirely exploratory in nature, with the aim of making suggestions for possible explanations of the study findings.

1. **Investigating differences between the open pilot study and the randomised controlled trial**

We observed that the pre-post differences for both the treatment and placebo arms were smaller in this trial than in the previous open-label pilot study. To investigate the potential reasons for this we looked at differences in the conduct of the pilot study and the trial and compared the estimated pre-post differences between the pilot study (CIAO-I) and the RCT (CIAO-II) at the HMP YOI Isis site only (since the pilot was only conducted at this site).

To investigate whether the pre-post differences were of different sizes in the pilot study (CIAO-I) and this trial CIAO-II), we applied similar inclusion and retention procedures across the two studies and restricted attention to HMP YOI Isis only. We obtained comparable data by applying similar compliance and follow-up rules to the OROS-MPH arm of the HMP YOI Isis data from this trial, as that applied in the pilot study. To match as closely as possible the procedure followed in the pilot study, participant outcome data from the current trial were treated as missing following a continuous period of 10 days without taking trial medication. To match the outcome period in the current trial (baseline to 8-week assessment) we used the 8-week assessment data from the pilot study (rather than the 12-week data) and only included participants who provided data at the 8-week timepoint.

Comparison of means are presented in Table 22. Table 23 presents a comparison of the demographic and baseline characteristics for participants in the pilot study and the current trial.

| **Table 16: Comparison of means for CAARS-O for pilot study and the subset of trial patients at HMP YOI ISIS taking OROS-MPH at least once in any consecutive period of 10 days** | | | | | | |
| --- | --- | --- | --- | --- | --- | --- |
| **Time** | **CIAO-I (pilot study)** | | | **CIAO-II (current trial)** | | |
|  | **N** | **Mean (SD)** | **Change from baseline** | **N** | **Mean (SD)** | **Change from baseline** |
| Baseline | 81 | 36.51 (6.91) |  | 40 | 37.63 (11.03) |  |
| Week 5 | 81 | 11.41 (8.17) | 25.10 | 41 | 25.49 (14.60) | 12.14 |
| Week 8 | 81 | 11.52 (8.77) | 24.99 | 41 | 26.55 (13.87) | 11.08 |

| **Table 17: Comparison of demographic and baseline measures for pilot study (CIAO-I) and the current trial (CIAO-II)** | | |
| --- | --- | --- |
|  | **CIAO-I**  **Total (n=121)** | **CIAOI-II (Isis only)**  **Total (n=115)** |
| Age [mean (sd)] | 21.09 (2.35) | 21.5 (1.9) |
| WASI-II IQ estimate [mean (sd)] | 90.48 (11.76) | 88.8 (12.3) |
| Education [Number (%)]  Above GCSE  GCSE  Other  Missing data | 2 (1.7)  33 (27.3)  69 (57.0)  17 (14.0) | 8 (7.0)  76 (66.0)  31 (27.0)  0 (0.0) |
| Employment [Number (%)]  In paid employment  Self-employed  Unemployed  Missing data | 12 (9.9)  1 (0.80)  103 (85.1)  5 (4.1) | 31 (27.0)  4 (3.5)  80 (70.0)  0 (0.0) |
| Ethnicity [Number (%)]  White  Asian  Black  Mixed  Other | 64 (52.9)  3 (2.5)  33 (27.3)  18 (14.9)  3 (2.5) | 40 (34.8)  2 (1.7)  46 (40.0)  22 (19.1)  5 (4.3) |
| Baseline symptom scales [mean (sd)]  CAARS-O  WRAADS emotional dysregulation  CGI  MVQ | 37.6 (7.0)  18.7 (5.3)  4.4 (0.6)  33.1 (10.0) | 39.0 (9.1)  18.3 (6.4)  4.7 (0.6)  32.1 (8.9) |
| Alcohol and drug use [N (%)]  Alcohol use  Cannabis use  Other drug use | 92 (78.0)  108 (91.5)  44 (37.3) | 74 (64.4)  109 (94.8)   1. 41.7) |

1. **Was the study outcome effected by a systematic change to trial procedures?**

To check whether a systematic change might have occurred during the duration of the at some time-point during the trial, such as unidentified changes in the conduct of the trial procedures, we provide outcomes by the order of randomisation into the trial. We provided outcome data by subgroups defined by order of recruitment: 1-50, 51-100, 101-150, and 151-200. As see in Table 24 there is little evidence to suggest that CAARS-O levels (across trial arms) varied between the four groups at any assessment time point. In addition, the sizes of the estimated between-trial arm effects on OROS-MPH at 8 weeks did not show any OROS-MPH effects for any of the four subgroups. By definition there are 50 participants in each sub-group and the estimated treatment difference between OROS-MPH and placebo arms, in chronological order of groups, is: 0.56 with 95% CI (-5.39 to 6.52), -0.001 with 95% CI (-6.41 to , 6.41), 3.45 with 95% CI (-3.90 to , 10.80), -0.54 with 95% CI (-5.99 to , 4.90).

| **Table 18: Descriptive summaries across trial arm** | **Group 1 (1-50)**  **Mean (sd)** | **Group 2 (51-100)**  **Mean (sd)** | **Group 3 (101-150)**  **Mean (sd)** | **Group 4 (151-200)**  **Mean (sd)** |
| --- | --- | --- | --- | --- |
| CAARS-O at baseline | 34.44 (9.72) | 33.96 (9.78) | 38.71 (9.31) | 40.16 (6.36) |
| CAARS-O at week 5 | 24.62 (13.31) | 28.16 (10.15) | 30.38 (12.38) | 29.68 (11.61) |
| CAARS-O at week 8 | 25.99 (12.43) | 28.54 (10.97) | 29.50 (13.02) | - 1. 10.02) |

1. **Was the study outcome affected by including less severe cases of ADHD in the trial?**

To check whether only the most severe cases benefit from drug treatment, we assessed the treatment effects in high and low severity groups for ADHD symptoms at baseline. We provided a subgroup analysis for high severity based on the baseline CAARS-O score, defined as t-scores of 70 (equivalent to a raw score of 35 or above). 123 participants had a baseline score of CAARS-O of 35 or greater. As shown in Table 25, this group show greater change scores than the overall trial: 12.26 for OROS-MPH, and 9.35 for placebo. This is an improvement in the OROS-MPH group compared to placebo of 2.81 (95% CI: -1.11 to 6.73, t=1.42, p=0.16) which is larger than for the overall trial, but does not reach the effect size specified in the sample size calculation (5 points) and is not statistically significant

| **Table 19: Subgroup for those with a CAARS-O raw score 35 or above** | **OROS-MPH**  **Mean (sd)** | **Placebo**  **Mean (sd)** | **Overall**  **Mean (sd)** |
| --- | --- | --- | --- |
| CAARS-O at baseline | 42.87 (4.93) | 42.63 (5.08) | 42.75 (4.98) |
| CAARS-O at week 5 | 30.39 (12.84) | 32.37 (11.12) | 31.40 (11.98) |
| CAARS-O at week 8 | 30.61 (11.92) | 33.28 (10.86) | 31.98 (11.42) |

1. **Was the study outcome affected by poor diagnostic accuracy in this prison population?**

To investigate the potential effect of diagnostic accuracy, we provide outcome data for a subgroup with a more clearly defined cluster of ADHD symptoms, using the DIVA diagnostic instrument data (Table 26). The rationale is that by raising the usual symptom count thresholds for the diagnosis, we will enhance the specificity of the symptom count data to the diagnosis of ADHD, thereby enhancing certainty for the diagnosis. This was achieved by defining as the sub-group, individuals meeting combined type criteria for ADHD in both childhood and adulthood from the DIVA assessment using symptom thresholds of 7 or more for both symptoms of inattention and hyperactivity-impulsivity, in both childhood and adulthood.

From the total sample of 200, 102 participants met these higher symptom thresholds. In this subgroup the estimated score difference between the OROS-MPH and placebo arms was estimated to be 1.83 (95% CI: -2.34 to 6.01, t = 0.87, p=0.39). This was a non-significant improvement in the OROS-MPH arm.

| **Table 20: Subgroup meeting higher threshold diagnostic criteria for ADHD** | **OROS-MPH**  **Mean (sd)** | **Placebo arm**  **Mean (sd)** |
| --- | --- | --- |
| AARS-O at baseline | 36.42 (9.76) | 37.18 (8.72) |
| CAARS-O at week 8 | 28.03 (11.86) | 29.33 (11.63) |

1. **Could the study outcome be affected by including participants with different levels of emotional dysregulation?**

Emotional dysregulation is known to be associated with ADHD and might reflect an index of severity of ADHD. However, symptoms of emotional dysregulation can also reflect the severity of other mental health disorders commonly seen in the prison population. It is therefore feasible that a group with high emotional dysregulation could show a greater effect of medication (if ADHD medication has greater effects in more severe cases) or might show a smaller effect of medication (if the symptoms of ADHD are better explained by a comorbid condition). We therefore investigated the outcomes by high and low sub-groups for the WRAADDS measure used as a baseline measure of emotional dysregulation (see Table 27).

The median WRAADDS score at baseline was 17 in the CIAO-II sample. The participants were roughly evenly divided between low and high emotional dysregulation with 113 participants having high emotional dysregulation. Table 24 shows summaries of the primary outcome variable by high or low emotional dysregulation and trial arm. The estimated score difference between the OROS-MPH and placebo arms in the subgroup with high emotional dysregulation was estimated to be 2.30 (95% CI: -1.81 to 6.41, t = 1.11, p=0.27). Although this is an improvement in the OROS-MPH arm, this is still below the difference stated in the sample size and is not statistically significant. This is an improvement in the OROS-MPH arm. There was also little observed difference between the two arms in the low emotional dysregulation subgroup with an estimated score difference between the OROS-MPH and placebo of -0.85 (95% CI: -5.54 to 3.84, t=0.36, p=0.72) which was an improvement in the OROS-MPH arm

| **Table 21: WRAADDS baseline severity compared to CAARS-O score** | **OROS-MPH arm** | | **Placebo arm** | |
| --- | --- | --- | --- | --- |
|  | **Low Emotional dysregulation**  **Mean (sd)** | **High Emotional dysregulation**  **Mean (sd)** | **Low Emotional dysregulation**  **Mean (sd)** | **High Emotional dysregulation**  **Mean (sd)** |
| CAARS-O at baseline | 33.03 (10.35) | 39.81 (7.88) | 33.69 (8.01) | 39.27 (8.51) |
| CAARS-O at week 5 | 25.27 (11.91) | 29.61 (13.26) | 27.52 (11.29) | 29.55 (11.61) |
| CAARS-O at week 8 | 26.69 (12.26) | 29.26 (11.47) | 26.26 (10.51) | 30.99 (11.95) |

1. **Could the study outcome be affected by including participants with borderline personality disorder?**

Symptoms of emotional dysregulation may also reflect the severity of other mental health disorders commonly seen in the prison population, such as borderline personality disorder. It can be difficult to separate out ADHD from borderline personality disorder on clinical grounds alone. For example, in our recent research we found that women meeting diagnostic criteria for borderline personality disorder also show high levels of inattentiveness in their daily lives, in addition to shared symptoms such as emotional dysregulation ^78^. To investigate the possibility that the sub-group meeting criteria for borderline personality disorder (BPD) might be reducing the overall effect of medication in the trial, we aimed to provide outcomes in sub-groups with and without borderline personality disorder. However, there were only 15 participants with high risk of BPD and 185 without. These analyses were not done as the BPD group was too small, and the low number of participants with BPD suggests this was not an issue and unlikely to provide an explanation.

1. **Could the study outcome be affected including participants with high levels of childhood trauma?**

The effect of childhood trauma has been widely discussed but has rarely been investigated as a potential modifier of ADHD medication treatment effects. In clinical practice some practitioners view childhood trauma as a potential cause of an ADHD-like syndrome that might not then show a typical response of ADHD symptoms to medication. To investigate the possibility that the sub-group with evidence of childhood trauma are reducing the overall effect of medication in the trial, we provide outcomes for subgroups with and without childhood trauma, defined as moderate to severe abuse according to the Childhood Trauma Questionnaire (CTQ) at baseline. The number of participants who reported abuse in the various categories are listed in Table 28. Out of 200 participants overall 41 reported emotional abuse, 47 reported physical abuse, 12 reported sexual abuse, 167 reported emotional neglect, and 197 reported physical neglect.

| **Table 22: CTQ sub-scale** | **OROS-MPH Mean (sd)** | **Placebo Mean (sd)** | **Overall Mean (sd)** |
| --- | --- | --- | --- |
| Emotional abuse | 9.05 (4.79) | 8.88 (5.07) | 8.97 (4.92) |
| Physical abuse | 8.07 (4.50) | 7.59 (4.55) | 7.83 (4.52) |
| Sexual abuse | 5.73 (3.10) | 5.47 (2.48) | 5.61 (2.81) |
| Emotional neglect | 20.22 (4.77) | 19.96 (5.28) | 20.09 (5.02) |
| Physical neglect | 13.35 (1.93) | 13.32 (1.94) | 13.34 (1.93) |

**Emotional abuse:** The estimated score difference between the OROS-MPH and placebo arm in the subgroup with high emotional abuse was 0.76 (95% CI: -4.67 to 6.18, t = 0.03, p=0.78). This is a small improvement in the OROS-MPH arm. The estimated score difference between the OROS-MPH and placebo arm in the subgroup without high emotional abuse was 1.20 (95% CI: -2.35 to 4.75, t=0.67, p=0.51), a small improvement in the OROS-MPH arm

**Physical abuse:** The estimated score difference between the OROS-MPH and placebo arm in the subgroup with high physical abuse was 1.62 (95% CI: -4.82 to 8.07, t = 0.51, p=0.61), a small improvement in the OROS-MPH arm. The estimated score difference between the OROS-MPH and placebo arm in the subgroup without high physical abuse was 0.85 (95% CI: -2.58 to 4.29, t = 0.49, p=0.62), a small improvement in the OROS-MPH arm

**Sexual abuse:** Due to the extremely small numbers, this statistical analysis was not done.

**Emotional neglect:** The estimated score difference between the OROS-MPH and placebo arm in the subgroup with high emotional neglect was -0.22 (95% CI (-3.13, 3.57)), (t = 0.13, p=0.90). This is a small improvement in the placebo arm.

In those without high emotional neglect the estimated score difference between the OROS-MPH and placebo arm was 3.71 with 95% CI (-3.26, 10.69) (t=1.09, p=0.29), which was an improvement in the OROS-MPH arm

**Physical neglect:** The statistical analysis was not done because 98.5% of the sample reported this.

1. **Could the study outcome be affected by including participants with comorbid disorders?**

One question raised by the trial is the possibility that comorbid disorders might reflect a source of symptoms and impairments that might mimic ADHD in the young male prison population. Including cases with clearly defined ‘co-morbid’ disorders might lead to reduction in the overall medication effect in the trial. To address this question, we provide subgroup data for those with and without evidence for another mental health disorder according to the MINI diagnostic interview for mental health disorders.

**Overall comorbidity:** 36 participants had no comorbidity. This subgroup was estimated to have a very small improvement in the OROS-MPH arm relative to placebo of 0.11 (95% CI: -7.23 to 7.45, t=0.03, p=0.98). 164 participants had at least one mental health comorbidity. The estimated score difference between the OROS-MPH and placebo arm for this subgroup was 0.98 (95% CI: -2.38 to 4.34, t=0.57, p=0.57). This is an improvement in the OROS-MPH arm but is not statistically significant and does not come close to the 5 points specified in the sample size calculation.

**Anxiety:** 162 participants did not have anxiety. For this subgroup, the estimated score difference between the OROS-MPH and placebo arm was 0.39 (95% CI: -2.82 to 3.60, t=0.24, p=0.81), which is a very small improvement in the OROS-MPH arm. 38 participants had an anxiety disorder. The estimated score difference between the OROS-MPH and placebo arm for this subgroup was 2.88 (95% CI: -5.68 to 11.43, t=0.69, p=0.50). This was an improvement in the OROS-MPH arm which, while larger than for the group without anxiety, is still not significant statistically.

**Mood disorder:** 137 participants did not have a mood disorder. For this subgroup the estimated score difference between the OROS-MPH and placebo arm was 0.32 (95% CI: -3.53 to 4.17, t=0.17, p=0.87), a very small improvement in the OROS-MPH arm. 63 participants had at a mood disorder. The estimated score difference between the OROS-MPH and placebo arm for this subgroup was estimated to be larger at 1.97 (95% CI: -3.14 to 7.09, t=0.77, p=0.44). This was a small improvement in the OROS-MPH arm but was not statistically significant.

**PTSD:** 13 participants had PTSD, 8 had depersonalisation, 4 had derealisation and 1 was not assessed for PTSD. The subgroup analysis was not done due to the extremely small subsamples.

**ASPD:** 51 participants did not have ASPD. The estimated score difference between the OROS-MPH and placebo arm in this subgroup was 0.98 (95%: CI: -7.06 to 5.09, t=-0.33, p=0.75), which was a small improvement in the OROS-MPH arm. 149 participants had at an ASPD. The estimated score difference between the OROS-MPH and placebo arm for this subgroup was 1.40 (95% CI: -2.18 to 4.98, t=0.77, p=0.44), which was a small improvement in the OROS-MPH arm. This was not statistically significant.

1. **Could the study outcome be affected by including participants with high levels of drug and alcohol?**

Drug and alcohol use disorders are both associated with ADHD and might have an impact on the treatment response. This could arise because of brain damage as a long-term consequence of drug and alcohol use either generating ADHD-like symptoms or reducing the potential effects of medication for ADHD. There could also be tolerance to OROS-MPH due to previous drug abuse which only normalises after a period. Further, a history of drug and alcohol abuse is likely to be an indicator for risk of ongoing (unmeasured) use of drugs within the prison during the trial, which might impact on ADHD symptoms. To address this question, we used the AUDIT-C data to identify participants with high risk of alcohol abuse, and the data from the NIDA screening tool to identify participants with high risk of drug abuse. We aimed to complete subgroup analyses for those at high and low risk for alcohol and drug abuse, to investigate whether including this group reduces the overall medication effects in the trial.

**High risk of problem alcohol use:** 89 people had potentially problematic alcohol use (defined as a score of 16 or higher on the AUDIT-C). The estimated score difference between the OROS-MPH and placebo arm in this subgroup was of 1.00 (95% CI: -3.84 to 5.85, t=0.41, p=0.68), a small improvement in the OROS-MPH arm. For those who did not report problematic alcohol use, the estimated score difference between the OROS-MPH and placebo arm was 1.88 (95% CI: -3.65 to 7.41, t=0.68, p=0.50). An improvement in the OROS-MPH arm.

**High risk of problem drug abuse:** Using the NIDA Quick Screen V1.0 criteria we classified individuals into low (none or minor use), medium, and high risk for substance involvement (see Table 26). We initially intended to apply the NIDA screening criteria for high risk of problematic drug, with a NIDA score of 27 or higher. However, although rates of reported drug use were high, the numbers meeting criteria for high risk of problem use were small, and for this reason these subgroup analyses were not conducted. Using the NIDA definition for high-risk use, there were only:

- 3 problem users of opiates (street or prescription). The minimum, median and maximum usage scores were 0, 0 and 32.
- 12 problem users of cannabis. The minimum, median and maximum usage scores were 0, 7 and 39.
- No problem users of ‘spice’. The minimum, median and maximum usage scores were 0, 0 and 17.
- 4 problem users of cocaine and methamphetamine. The minimum, median and maximum usage scores were 0, 0 and 39.

Because of the low numbers in the high-risk groups, we decided to complete an additional analysis regarding drug use to address the question of whether a group free of either moderate or severe use of any drug (score of 4 or higher on the NIDA) showed a difference in the CAARS-O outcome at 8 weeks for the OROS-MPH versus placebo arms.

73 participants had no or very low use of any of opiates, spice, cannabis, or stimulants. In this subgroup, the estimated score difference between the OROS-MPH and placebo arm was 4.26 (95% CI: -9.31 to 0.78, t=-1.69, p=0.10). The OROS-MPH arm did better. See appendix 7 for more details regarding illicit drug usage prior to incarceration.

| **Table 23: NIDA scores for each drug class** | **NIDA risk group** | **OROS-MPH N (%)** | **Placebo N (%)** | **Overall N (%)** |
| --- | --- | --- | --- | --- |
| Opiates | Low | 97 (96.0) | 96 (97.0) | 193 (96.5) |
|  | Medium | 2 (2.0) | 2 (2.0) | 4 (2.0) |
|  | High | 2 (2.0) | 1 (1.0) | 3 (1.5) |
| Cannabis | Low | 40 (39.6) | 41 (41.4) | 81 (40.5) |
|  | Medium | 55 (54.5) | 52 (52.5) | 107 (53.5) |
|  | High | 6 (6.0) | 6 (6.0) | 12 (6.0) |
| Spice | Low | 100 (99.0) | 95 (96.0) | 195 (97.5) |
|  | Medium | 1 (1.0) | 4 (4.0) | 5 (2.5) |
|  | High | 0 (0.0) | 0 (0.0) | 0 (0.0) |
| Stimulants | Low | 81 (80.2) | 88 (88.9) | 169 (84.5) |
|  | Medium | 17 (16.8) | 10 (10.1) | 27 (13.5) |
|  | High | 3 (3.0) | 1 (1.0) | 4 (2.0) |
| Total | Low | 39 (38.6) | 34 (34.3) | 73 (36.5) |
|  | Medium | 54 (53.5) | 57 (57.6) | 111 (55.5) |
|  | High | 8 (7.9) | 8 (8.1) | 16 (8.0) |

1. **Could the study outcome be affected by including participants with prior experience of stimulant medication leading to biased reporting of the ADHD symptom response to medication?**

Prior exposure to stimulant treatment for ADHD could change the reporting of symptoms because of prior experience of effects of medication on ADHD symptoms and adverse effects, leading to biased reporting. There is also the possibility that medication was stopped in these cases because of previous non-response or significant adverse effects. To investigate the possibility that these effects might reduce the overall effect of medication in the trial, we conducted sub-group analyses for groups with and without previous exposure to stimulant medication for ADHD.

153 had no previous treatment with stimulants for ADHD and 1 person was not assessed. 46 people had received stimulants for ADHD in the past. The subgroup who had not previously been treated with stimulants for ADHD had an estimated score difference between the OROS-MPH and placebo arm of 0.63 (95% CI: -2.93 to 4.19, t=-0.35, p=0.72), and the OROS-MPH arm did better. 46 participants had received stimulants for ADHD in the past with the last dose taken at least 3 months earlier. The estimated score difference between the OROS-MPH and placebo arm for this subgroup was 2.01 (95% CI: -4.17 to 8.19, t=0.66, p=0.51), a small improvement in the OROS-MPH arm which was not statistically significant.

1. **Could the study outcome be affected by under-dosing of participants?**

The optimal dose of methylphenidate used in the treatment of adult ADHD is highly variable, hence we adopted a titration procedure to individually titrate each participant to the optimal dose of study medication. However, as noted in the pilot CIAO-I study, this population were concerned by minor adverse effects and were titrated to relatively low average doses of the trial medication. This suggests that in this population the titration protocol might lead to under dosing of participants, potentially accounting for lower effects of drug versus placebo. To investigate this possibility, we compared the study outcome for subgroups titrated to a low dose versus a high dose, defined as sub-groups titrated to 1 or 2 capsules (low dose), and those titrated to 3 or 4 capsules (high dose).

Table 10 shows the distribution of the prescribed dose at the start of week-5 and at the start of week-6 by trial arm (see section 5.7 for explanation of week-6 prescriptions) for those who were ongoing in the trial. As noted in the primary analyses those allocated OROS-MPH were titrated to lower doses than those in the placebo arm. For example, at the start of week-5, 74% were prescribed the higher dose (3 or 4 capsules) in the OROS-MPH arm, compared to 86% in the placebo arm.

|  |
| --- |

| **Table 24: The number of prescribed capsules at the start of week 5 and week 6 for those ongoing in the trial** | | | |
| --- | --- | --- | --- |
| Number of capsules prescribed per day | OROS-MPH  N (%) | Placebo  N (%) | Total sample  N (%) |
| **Start of week 5** | | | |
| 1 | 9 (11.0) | 3 (3.2) | 12 (6.8) |
| 2 | 11 (13.4) | 10 (10.5) | 21 (11.9) |
| 3 | 21 (25.6) | 25 (26.3) | 46 (26.0) |
| 4 | 40 (48.8) | 57 (60.0) | 97 (54.8) |
| Not prescribed | 1 (1.2) | 0 (0.0) | 1 (0.56) |
| **Start of week 6** | | | |
| 1 | 7 (9.0) | 2 (2.2) | 9 (5.3) |
| 2 | 10 (12.8) | 10 (10.8) | 20 (11.7) |
| 3 | 19 (24.4) | 19 (20.4) | 38 (22.2) |
| 4 | 42 (53.9) | 62 (66.7) | 104 (60.8) |

At the start of week 5, 143 participants were prescribed 3 or 4 tablets. The estimated score difference between the OROS-MPH and placebo arm in this subgroup was 0.47 (95% CI: -3.03 to 3.97, t=-0.27, p=0.79), which was an improvement in the OROS-MPH arm. 57 participants were prescribed 1 or 2 tablets at the start of week 5. The estimated difference between OROS-MPH vs placebo arms within this subgroup was 0.68 (95% CI: -6.15 to 7.51, t=0.20, p=0.84), which is a small improvement in the OROS-MPH arm that is not statistically significant.

1. **Could the study outcome be affected by poor adherence to trial medication?**

Adherence to taking the trial medication on a daily basis was highly variable and might therefore have impacted the assessment of efficacy by ITT. The high levels of poor adherence with medication was previously documented in the CIAO-I pilot study where 40 out of 121 (33.9%) participants dropped out of treatment before the 8-week assessment, and efforts were therefore taken in the current trial to minimise this ^29^. The per protocol analysis carried out according to our SAP defined “compliance” for the trial medication, as having taken medication on 75% of the day’s during the trial period, did not however show any different effect from the ITT analysis, suggesting that poor compliance is unlikely to explain the study findings. Nevertheless, it is possible that our definition of compliance was not adequate to rule out all effects of adherence to the study medication on the study outcome. To address this problem, we investigated the impact of a set of alternative definitions of adherence with the trial mediation on efficacy in this trial.

To provide an overview of the amount of drug prescribed and taken, we produced descriptors of the dose prescribed and dose taken. This is already reported as part of the main results for those ongoing on medication and using the prescription data from the start of week 5 of the trial.

The protocol also allowed a final dose change at the start of week 6, prior to the final 3 week maintenance phase of the trial. Note that for weeks 1 to 5 the prescription was explicitly recorded in the database. For week 6 the prescription was not recorded but is inferred from the maximum dosage taken by participants. In all cases, the nurses providing the prescriptions and recorded a daily log of the dose taken. It is therefore reasonable to infer a prescription change for week 6 from the maximum dose taken in days 36, 37, 38 and 39, which is in line with the protocol for week 6 prescriptions. Using this approach, we describe the number of participants prescribed different doses of trial medication from the start of week 6 (see Table 27) which was the maintained until the end of the trial. Table 28 shows the numbers recorded as prescriptions for week 1 to 5 and the inferred prescription for week 6.

| **Table 25: Mean daily tablets prescribed by week of those who were continuing treatment** | | | | |
| --- | --- | --- | --- | --- |
| **Week** | **Number of participants prescribed trial medication** | **OROS-MPH**  **(N=101)** | **Placebo**  **(N=99)** | **Overall**  **(N=200)** |
|  | **N (%)** | **Mean (s.d.) numbers of tablets prescribed** | | |
| Week 1 | 200 (100.0) | 1.00 (0.00) | 1.00 (0.00) | 1.00 (0.00) |
| Week 2 | 197 (98.5) | 1.67 (0.47) | 1.71 (0.46) | 1.69 (0.46) |
| Week 3 | 191 (95.5) | 2.24 (0.74) | 2.44 (0.67) | 2.35 (0.71) |
| Week 4 | 183 (91.5) | 2.80 (0.98) | 3.06 (0.96) | 2.7 (0.71) |
| Week 5 | 177 (88.5) | 3.14 (1.03) | 3.41 (0.84) | 3.28 (0.94) |
| Week 6 | 171 (85.5) | 3.20 (1.03) | 3.49 (0.81) | 3.36 (0.92) |

To describe the overall adherence with prescribed trial medication, we describe the mean daily capsules taken by week and percentage of prescribed dosage taken (Table 29). Prescriptions for weeks 1 to 5 are based on the recorded prescription. The prescription for week 6 is inferred from the maximum tablets taken. The prescription for weeks 7 and 8 is identical to week 6. Note that by the final week, the data is skewed, it is however the percentage of medication taken of that prescribed (documented or inferred) that is of interest. Throughout the trial, the placebo group took a larger percentage of the tablets prescribed. By week 4 this difference had become more marked and remained more marked for the remaining weeks.

| **Table 26:** **Mean daily tablets taken by week and percentage of prescribed dosage taken** | | | | | | |
| --- | --- | --- | --- | --- | --- | --- |
| **Week** | **OROS-MPH**  **(N=101)** | | **Placebo**  **(N=99)** | | **Overall**  **(N=200)** | |
|  | **Mean (s.d.) capsules taken by week** | **Percentage of prescribed medication** | **Mean (s.d.) capsules taken week** | **Percentage of prescribed**  **medication** | **Mean (s.d.) capsules taken week** | **Percentage of prescribed medication** |
| Week 1 | 0.76 (0.32) | 76.0 | 0.86 (0.23) | 86.0 | 0.81 (0.28) | 81.0 |
| Week 2 | 1.23 (0.69) | 73.7 | 1.35 (0.60) | 78.9 | 1.29 (0.65) | 76.3 |
| Week 3 | 1.49 (0.98) | 66.5 | 1.89 (0.88) | 77.5 | 1.69 (0.95) | 71.9 |
| Week 4 | 1.55 (1.22) | 55.4 | 2.22 (1.20) | 72.5 | 1.88 (1.25) | 69.6 |
| Week 5 | 1.61 (1.38) | 51.3 | 2.40 (1.27) | 70.4 | 2.00 (1.38) | 61.0 |
| Week 6 | 1.57 (1.38) | 49.1 | 2.33 (1.32) | 66.8 | 1.95 (1.40) | 58.0 |
| Week 7 | 1.59 (1.38) | 49.7 | 2.25 (1.43) | 64.5 | 1.92 (1.44) | 57.1 |
| Week 8 | 1.41 (1.34) | 44.1 | 1.99 (1.41) | 57.0 | 1.70 (1.40) | 50.6 |

Figure 6 Illustrates the mean adherence of participants by trial arm. The weekly adherence is the defined as the number of days in which at least one tablet was taken divided by seven. At the start of the trial the mean adherence was 71.6% and 81.5% in the OROS-MPH and placebo arms. This dropped to 50.6% and 70.4% in week five and 43.7% and 56.6% by week eight. The proportion of people adhering in the placebo group was consistently higher throughout the trial but adherence drops off consistently throughout the trial in both arms.

**Figure 6: Mean adherence with prescribed trial medication**

Adherence to trial medication might have been higher during the titration phase of the trial (when participants were visited every week) than during the maintenance phase. To investigate this, we conducted a per protocol analysis of the effects of OROS-MPH versus placebo on the CAARS-O outcome at week 5. Good compliance during this period was defined as those who had taken prescribed trial medication on 75% of the days before the end of week 5.

According to this definition, 88 participants who had complied with their allocated drug by week 5 (taken at least one tablet on 75% of the days up to and including day 35) there was a between arm difference in CAARS-O score between the OROS-MPH and placebo arms of -1.32 95% CI (-6.14 to 3.50, t=-0.54, 0.59), which is an improvement in the placebo arm. The mean and sd by trial arm in this group is shown in Table 30.

| **Table 27: Summaries of mean CAARS-O scores at 5 weeks for the subgroup with good compliance in the first 5 weeks of the trial** | | |
| --- | --- | --- |
| CAARS-O | OROS-MPH  Mean (sd) | Placebo  Mean (sd) |
| Baseline | 35.8 (9.4) | 36.3 (8.4) |
| Week 5 | 28.3 (12.3) | 27.0 (11.6) |

Adherence for trial medication immediately prior to the outcome assessments might also affect the study outcome, even if the active medication was significantly reducing ADHD symptoms on days when they did take the medication. This could occur because participants might only report accurately on their level of ADHD symptoms on the day or the few days prior to the outcome assessment. The short term-effects of OROS-MPH on reducing ADHD symptoms (for only 6-10 hours) means that participants in the active medication group might report high levels of symptoms if they had not taken medication in the period just prior to the outcome assessment; even in those that showed an effect on days when they did take the medication. To investigate the immediate effects of the trial medication, we conducted a further per protocol analyses on the week 5 and week 8 CAARS-O data. Adherence was defined as taking trial medication on the two days prior to the outcome assessment at week 5 and week 8, with those not taking medication on those days excluded from the analysis.

For week 5 there were 97 participants who had taken one or more capsules on both days immediately preceding. There was an estimated difference between the OROS-MPH arm and placebo in this subgroup of 2.30 (95% CI: -2.28 to 6.88, t=1.00, p=0.32) which was an improvement in the OROS-MPH arm. For week 8 within the subgroup of 84 participants (one of whom did not provide an outcome) who had taken at least one capsule on both of the days preceding the week 8 visit, the estimated difference between the OROS-MPH and placebo arms was -0.22 (95% CI: -4.87 to 4.44, t=-0.09, 0.93, which was a small improvement in the placebo arm.

1. **Could adverse effects be driving adherence to the trial medication?**

To investigate the potential association of adverse effects on adherence to taking the trial medication we investigated the proportion of prescribed medication taken by the presence or absence of the most commonly reported adverse effects of OROS-MPH versus placebo. We used the AES data from across the trial. As can be seen in Table 18, the common items from the AES showing the greatest difference between the two trial arms were sweating, headache, appetite loss and dry mouth. These were reported by 16%, 18%, 16% and 30% of participants respectively, and 50% of participants reported one or more of these.

For this analysis we investigated the adherence to medication for each of these 4 side AES items, as follows: (1) We measured adherence as the proportion of days on which any dose was taken divided by number of days medication was prescribed; (2) we calculated the mean proportion for groups reporting the presence or absence of each of the 4 AES items, and ran t-tests to compare the present versus absent groups; (3) we also considered an overall AES item measure of having at least one of the 4 items.

In Table 31, mean adherence is shown by groups where the AES items were present or not. A difference in mean proportion of adherence by symptoms was tested using a t-test. There was no evidence to suggest that the adherence varied between those who did and did not display the most common adverse effects.

| **Table 28: Adherence to medication** | | | |
| --- | --- | --- | --- |
| AES reported adverse effect | Proportion of days  mean (sd) | | Two-sided t-test |
|  | AE reported – YES | AE reported - NO | P-value |
| Headache | 0.58 (0.24) | 0.67 (0.25) | 0.44 |
| Dry mouth | 0.68 (0.22) | 0.65 (0.26) | 0.81 |
| Sweating | 0.62 (0.25) | 0.66 (0.25) | 0.36 |
| Appetite loss | 0.66 (0.23) | 0.65 (0.26) | 0.88 |
| Any | 0.66 (0.23) | 0.65 (0.27) | 0.81 |

`
